# In silico analysis of R2R3-MYB transcription factors in the basal eudicot model, *Aquilegia coerulea*

**DOI:** 10.1007/s13205-024-04119-y

**Published:** 2024-10-29

**Authors:** Banisha Phukela, Hanna Leonard, Yuval Sapir

**Affiliations:** 1 The Botanical Garden, School of Plant Sciences and Food Security, Faculty of Life Sciences, Tel Aviv University, Tel Aviv, Israel; 2 Department of Botany, Miami University, Oxford, OH 45056 USA

**Keywords:** Gene duplications, ABP pathway, Phylogeny, C-termini motifs, Subgroup 6

## Abstract

R2R3-MYBs are an important group of transcription factors that regulate crucial developmental processes across the plant kingdom; yet no comprehensive analysis of the R2R3-MYBs in the early-diverging eudicot clade of Ranunculaceae has been conducted so far. In the present study, *Aquilegia coerulea* is chosen to understand the extent of conservation and divergence of R2R3-MYBs as a representative of the family by analysing the genomic distribution, organization, gene structure, physiochemical properties, protein architecture, evolution and possible mode of expansion. Genome-wide analysis showed the presence of 82 putative homologues classified into 21 subgroups, based on phylogenetic analysis of full-length protein sequences. The domain has remained largely conserved across all homologues with few differences from the characterized *Arabidopsis thaliana* R2R3-MYBs. The topology of the phylogenetic tree remains the same when full-length protein sequences are used, indicating that the evolution of R2R3-MYBs is driven by the domain region only. This is supported by the presence of similar structures of exon–intron and conserved motifs within the same subgroup. Furthermore, comparisons of the AqcoeR2R3-MYB members with monocots and core-eudicots revealed the evolutionary expansion of a few functional clades, such as *A. thaliana* R2R3-MYB subgroup 6 (SG6), the upstream regulatory factors of floral pigment biosynthesis and floral color. The reconstructed evolutionary history of SG6-like genes across angiosperms highlights the occurrence of independent duplication events in the genus *Aquilegia*. AqcoeR2R3-MYB genes are present in all seven chromosomes of *A. coerulea*, most of which result from local and segmental duplications. Selection analysis of these duplicated gene pairs indicates purifying selection except one, and the physiochemical analyses of R2R3-MYBs reveal differences among the MYBs signifying their functional diversification. This study paves the way for further investigation of paralogous copies and their probable role in the evolution of different floral traits in *A. coerulea*. It lays the foundation for functional genomic studies of R2R3-MYBs in the basal eudicots and facilitates comparative studies among angiosperms. The work also provides a framework for deciphering novel genetic regulatory pathways that govern the diversity of floral morphology.

**Supplementary Information:**

The online version contains supplementary material available at 10.1007/s13205-024-04119-y.

## Introduction

R2R3-MYBs form the largest family of transcription factors in plants and are characterized by the presence of an evolutionarily conserved MYB domain at their N-terminal region (Dubos et al. 2010). The MYB domain consists of two repeats i.e., R2 and R3, with each repeat of about 52 amino acids forming a helix-loop-helix-turn-helix (HTH) structure (Stracke et al. 2001). The N-terminal MYB domain mediates the interaction with downstream target DNA, while the C-terminus varies across different R2R3-MYB members with altogether diverse regulatory roles (Jiang et al. 2004; Kranz et al. 1998; Katiyar et al. 2012). R2R3-MYBs are known to influence the transcription of many downstream target genes that control crucial biological processes in plants such as biotic and abiotic stress, secondary metabolism, development and differentiation (Wu et al. 2022).

R2R3-MYBs were initially identified and characterized in the model plant, *Arabidopsis thaliana* (L.) Heynh. (Kranz et al. 1998). Most of the members from this gene family resolved into 22**–**25 phylogenetically distinct and well-supported subgroups based on their sequence homology, the presence of conserved motifs at C-termini, the positions of conserved introns, expression patterns and functions; few R2R3-MYB genes remained orphan with unknown functions (Kranz et al. 1998; Stracke et al. 2001; Lal et al. 2022). Subsequently, R2R3 gene family members were identified and characterized in other sequenced plant model systems from monocots and core-eudicots (Cao et al. 2013; Liu et al. 2014; Matus et al. 2008; Wilkins et al. 2009). Most members are conserved and usually fall into the same phylogenetic subgroups as in *A. thaliana*, with few selective lineage-specific expansions and divergences observed across species. This strong conservation emphasizes the importance and ubiquity of this transcription factor family during the course of evolution (Feng et al. 2015; Huang et al. 2018; Li et al. 2022; Pucker et al. 2020; Wang et al. 2020a). Nonetheless, these R2R3-MYB genes have rarely been studied across the basal eudicots. Here we utilized the sequenced genome of *Aquilegia coerulea* E. James, a basal eudicot from Ranunculaceae, to understand the evolution of the R2R3-MYB gene family.

Members of the early-diverging eudicot family Ranunculaceae are well-known for the presence of diverse floral phenotypic traits, ranging from extremely primitive to highly derived ones (Hoot 1991; Tamura 1995). Amongst them, the genus *Aquilegia* L. is particularly interesting owing to taxa that show abundant morphological, ecological, and geographical diversity with little observed genetic differences (Hodges and Arnold 1994; Hodges 1997; Whittall and Hodges 2007; Jabbour et al. 2009). Aquilegias have previously been used in a number of evolutionary and developmental analyses to understand the origin of floral traits such as petaloid sepals, additional whorls of staminodia and nectar spurs (Kramer 2009). All of these studies highlighted the involvement of transcriptional factors which underwent few duplication events, followed by sub- or neo-functionalization in *Aquilegia* that might have facilitated the evolution of novel organs in the flowers (Sharma et al. 2011; Sharma and Kramer 2013; Meaders et al. 2020).

*Aquilegia* exhibits remarkable diversity in floral forms, such as petaloid organs of different origin, color (including blue, purple, red, yellow, green, and white), size, orientation, and cell shapes (Kramer 2009; Kramer and Hodges 2010). These traits have facilitated the rapid diversification of species (Hodges 1997; Kay et al. 2006; Whittall and Hodges 2007). From the perspective of pollination, flower color serves as a cue for attraction, and is strongly associated with different pollination syndromes, besides promoting speciation events (Rausher 2008). Even more so, floral color has piqued the attention of garden enthusiasts and scientists engaged in producing horticultural varieties (Hodges 2015). The other adaptation shown by aquilegias is that they can grow in diverse habitats ranging from damp forests, springs, to alpine zones (Munz 1946). Current knowledge on the genetic basis of these diverse floral and vegetative traits is limited. Therefore, it is important to shed light on one of the most abundant groups of transcription factor families, i.e., R2R3-MYBs, which may identify candidate genetic changes associated with traits. In the present work, the Ranunculaceae model system *Aquilegia coerulea* has been chosen to understand the extent of conservation and divergence of important R2R3-MYB transcriptional factors in the species. Furthermore, *Aquilegia* holds an important phylogenetic position, as it is a representative of Ranunculaceae which is sister to the rest of the core-eudicots. This will allow us to make evolutionary comparisons with both monocots and core-eudicots (APG IV, Angiosperm Phylogeny Group, 2004).

There are numerous instances of clade-specific gene duplications reported in this gene family contributing to the evolution of novel traits in angiosperms (Hui et al. 2019; Lin and Rausher 2021; Muñoz-Gómez et al. 2021; Wang et al. 2023). Usually, the mechanisms responsible for gene family expansions are whole genome duplications (WGD), segmental, or local duplication events (Ohno 1970). When these events are accompanied by neo-functionalization, sub-functionalization or pseudogenization, they serve as drivers of evolutionary innovations. The present manuscript provides a genome-wide analysis of R2R3-MYB transcription factors in an early-diverging eudicot species, *A. coerulea*. The study aims to identify and catalogue all R2R3-MYB members present in its genome. The study further examines the structural features of each gene, and their conserved protein domains, chromosomal distribution, protein architecture, selection pressure, duplication events and phylogenetic relationship among its members. This is the first exploration of this gene family in one of the model species of Ranunculaceae. The detailed study on R2R3-MYBs in the species serves to bridge the gap between monocots and core-eudicots due to its unique phylogenetic position. The study revealed important insights into the novel genic and protein features and highlighted the selective expansion of a few subgroups related to diverse floral color production and male gamete formation. The study might help us to understand the evolution of R2R3-MYBs in the basal eudicot family and provide valuable candidate genes for future functional analysis.

## Materials and methods

### Identification of putative R2R3-MYB homologues in *A. coerulea*

126 R2R3-MYB protein sequences of *A. thaliana* from The *Arabidopsis* Information Resource (TAIR, http://www.arabidopsis.org/), and two additional R2R3-MYB sequences, *AtMYB125* (*DUO1*; *AT3G60460*) (Higo et al. 2018) and *DIVARICATA2* (*DIV2*; *AT5G04760*) (Fang et al. 2018) were acquired. All 128 *A. thaliana* sequences were used as query to identify putative orthologues in the *A. coerulea* v3.1 genome in the plant comparative genomic portal database (Filiault et al. 2018; Phytozome, https://phytozome-next.jgi.doe.gov/). Initially, TBlastN searches were performed by keeping default parameters on (expect threshold -1, compositional matrix BLOSUM62, word length default, allow gaps, filter query). The resultant blast hits were confirmed for the presence of the R2R3-MYB domain in their protein sequences using Pfam (http://pfam.xfam.org/) and PROSITE databases (https://prosite.expasy.org) based on criteria used by Lal et al. (2021). Sequences identified with duplicate identities and with partial R2R3-MYB domain were removed based on analysis.

### R2R3-MYB domain sequence analysis

Amino acid sequences of R2 and R3 repeats of all *A. coerulea* homologues were aligned using MEGA X v10.2.6 (Kumar et al. 2018) and manually adjusted. The multiple sequence alignment (MSA) output files were submitted to the web-logo server to create sequence logos for both R2 and R3 repeats, separately (https://weblogo.berkeley.edu/, v2.8.2, Crooks et al. 2004).

### Phylogenetic reconstruction and conserved motifs of AqcoeR2R3-MYBs

To understand the evolutionary history of R2R3-MYBs in *A. coerulea*, protein sequences of all homologs were aligned using the Muscle program in MEGA-X v10.2.6 (Kumar et al. 2018) keeping default parameters on. The MSA output file generated was used to find the best substitution model. A neighbor-joining (NJ) tree was constructed with the following settings: substitution model, LT; test of phylogeny, 1000 bootstrap replicates; rate among site, 4; and gap/missing data treatment, pairwise deletion. Similarly, to further predict the function of novel genes from *A. coerulea*, R2R3-MYB sequences from *A. thaliana*, *Oryza sativa*, *Vitis vinifera*, *Populus trichocarpa* and some other eudicots were also included in the analysis (Supplementary Table 1). A total of 689 R2R3-MYB full-length protein sequences were aligned in MEGA-X v10.2.6 and a NJ tree was constructed with the following settings: substitution model, LT; test of phylogeny, 1000 bootstrap replicates; rate among site, 4; and gap/missing data treatment, pairwise deletion.

The conserved motifs outside the R2R3-MYB domain regions were predicted using MEME suite 5.5.2 (Motif-based sequence tool, https://meme-suite.org/meme/tools/meme, Bailey et al. 2015) based on parameters selected as discovery mode: classic, site distribution: zero or one occurrence per sequence, number of motifs to find: 40, motif width range from 6 to 100. The motifs were visualized with TBtools software (Chen et al. 2020).

### Structural and physicochemical properties

To understand the exon–intron distribution pattern, genomic sequences and coding sequences (CDS) of all putative AqcoeR2R3-MYB homologues were downloaded. Both genomic and coding sequences for each homologue were submitted to the Splign alignment tool (Kapustin et al. 2008). The tool aligned each transcript sequence to its respective genomic sequence and predicted a gene model specifying exon–intron boundaries, frameshifts and also splice junctions, if present. The sequences also included UTR regions wherever annotated and available. The predicted gene model and intron phases were displayed through the gene structure display server (GSDS 2.0, Hu et al. 2015). Furthermore, molecular weight and isoelectric point were calculated through the expasy server (https://web.expasy.org/compute_pi/). GRAVY values (Grand Average of Hydropathy) were analysed through a sequence manipulation suite (SMS; https://www.bioinformatics.org/sms2/protein_gravy.html).

To predict the tertiary structure, the amino acid sequence of domain regions for each homolog was submitted to Phyre 2.0 protein modelling server to search for evolutionary and structurally related template structures (http://www.sbg.bio.ic.ac.uk/phyre2/html/page.cgi?id=index). The output generated shows templates ranked in order of alignment coverage, confidence, % id and template information. The model with the highest coverage and % id was selected as the best model for which the PDB file was downloaded and edited in UCSF Chimera X V1.1 (Pettersen et al. 2021). The ancestral sequence reconstruction (ASR) of the R2R3-MYB domain based on 82 AqcoeR2R3-MYB extant homologous protein sequences was also performed using the Maximum Likelihood method as implemented in MEGA X v10.2.6 (Kumar et al. 2018). Ancestral sequences for each node across the phylogeny were retrieved with the help of a utility program ExtAncSeqMEGA (Kumar et al. 2018). However, the ancestral protein sequence of nodes with bootstrap-supported subgroups (SGI-SGXXI) and ancestral sequence at root were only proceeded for secondary and tertiary structure prediction on Phyre 2.0 protein modelling server. Deviation in the secondary structure of AqcoeR2R3-MYBs was evaluated using a matchmaker tool in Chimera by superimposing extant proteins over the ancestral protein structure.

## Genomic distribution and duplication analysis of R2R3*-*MYB genes in *A. coerulea*

The genomic coordinates were retrieved for all putative homologues to locate their positions on chromosomes using MapChart software (Voorrips 2002, https://www.wur.nl/en/show/Mapchart.htm). To infer possible relationships between R2R3-MYB genes, the occurrence of local and segmental duplications was analysed within the *A. coerulea* genome. Gene duplications were predicted based on sequence coverage (> 60% throughout the protein and > 90% within the MYB domain region) and phylogenetic relationships. The paralogues confirmed for local and segmental duplications shown on the physical map of chromosomes using MapChart software and also visualized using Circos tool (Cheong et al. 2015). Furthermore, Ka/Ks substitution values for *A. coerulea* duplicated gene pairs were calculated to infer their mode of selection (Zhang 2022).

### Phylogenetic analyses of SG6 genes

Initially, protein sequences of canonical members of SG6 R2R3-MYB genes from *A. thaliana* (AtMYB75, AtMYB90, AtMYB113, and AtMYB114) were downloaded and used as queries to identify putative homologs against scaffold-level assembly of *A. eximia* and *Thalictrum thalictroides* and chromosome-level assembly of *A. kansuensis* and *Coptis chinensis* (https://www.ncbi.nlm.nih.gov/). The sequences retrieved after the tBLASTn searches with default parameters were translated to find the open reading frames (ORFs) so as to keep only the coding sequences in the analysis. Other SG6 putative homologues included in the analysis outside Ranunculaceae were obtained from public databases such as NCBI (https://www.ncbi.nlm.nih.gov/), Phytozome (https://phytozome-next.jgi.doe.gov/) and SolGenomics (https://solgenomics.net/). The list of sequences used in the analysis is provided in Supplementary Table 2. A total of 121 R2R3-MYB full length protein sequences were aligned using ClustalW with general parameters and output format as FASTA (https://www.genome.jp/tools-bin/clustalw, Thompson et al. 1994). The MSA file generated was used to construct a maximum likelihood (ML) based phylogeny using IQ-tree (Cipres science gateway, http://www.phylo.org/). For branch support, bootstrap of 1000 pseudo-replicas was also implemented in IQ-TREE (Hoang et al. 2018). The tree output file obtained was visualized and edited using FigTree v1.4.4 (http://tree.bio.ed.ac.uk/software/figtree/, Rambaut and Drummond 2012).

## Results and discussion

### Identification and conserved DBD analysis of AqcoeR2R3-MYBs

Blast analyses of 128 AtMYBs against *A. coerulea* v3.1 genome on the phytozome database and subsequent gene prediction of retrieved sequences revealed the presence of 82 members of the R2R3-MYB gene family (Supplementary Table 3). All members showed two MYB repeats mainly on the N-terminal region and were mapped to unique loci in the genome. The genes possessing partial repeat regions were excluded from the study. The identified R2R3 members are named AqcoeMYB1 to AqcoeMYB82, based on the order of their identified locations on the chromosomes (Supplementary Table 3).

**Fig. 1:**
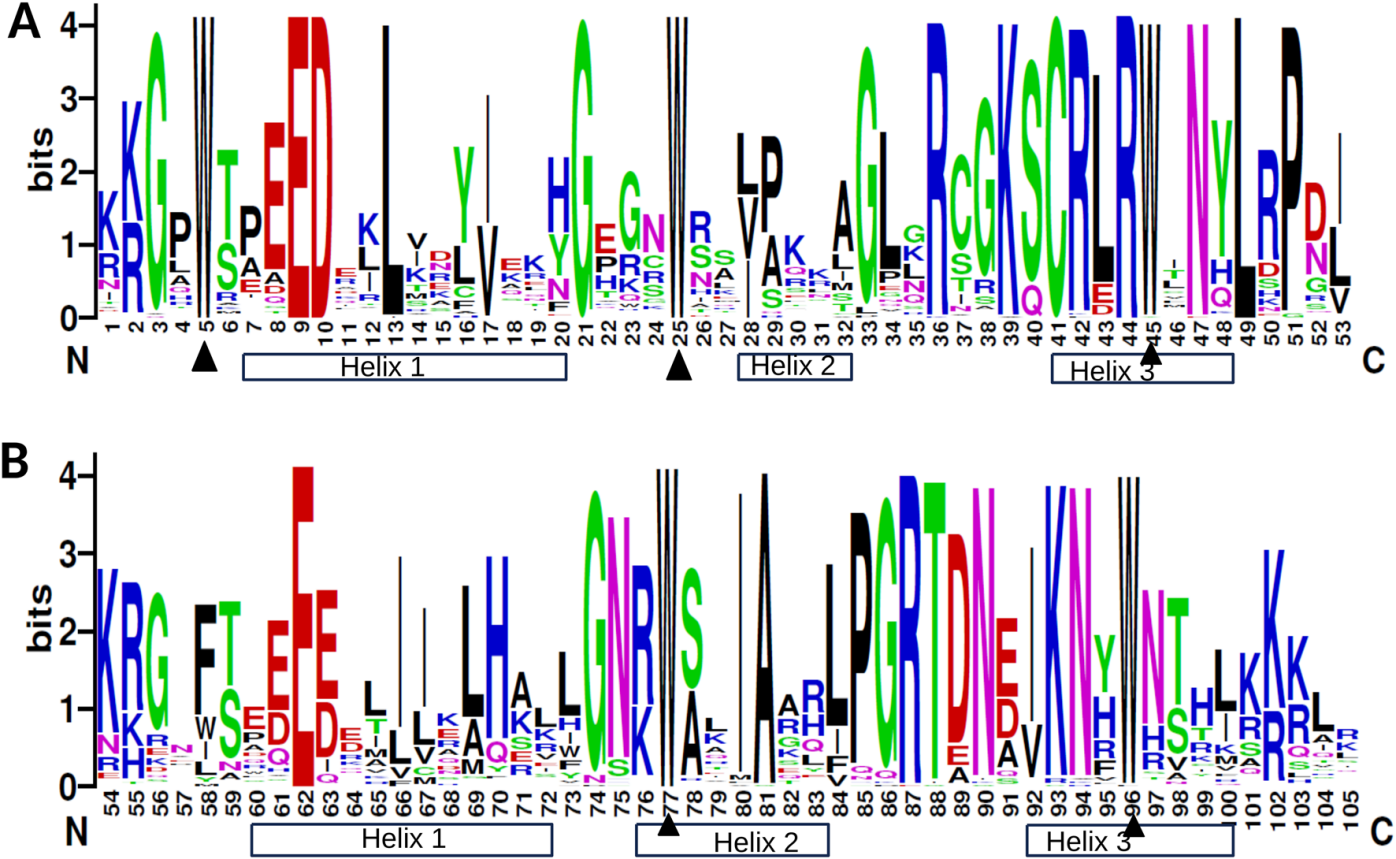
Sequence logos of *A. coerulea* R2R3-MYB domain: Sequence logos of R2 (**A**) and R3 (**B**) repeat regions based on full-length alignment of MYB domain showing highly conserved residues present across all R2R3-MYB proteins in *A. coerulea*. The height of stack represents sequence conservation at that position while bit score represents the frequency of that particular residue at that position in the sequence. The regions involved in helix formation is shown in rectangles whereas triangles depict the highly conserved Trp (W) residues in the MYB domain

MSA across all identified homologues was performed and sequence logos were created for both R2 and R3 repeats, separately to understand the sequence features and degree of conservation of each residue of the MYB domain (Fig. 1, Supplementary Table 3). Usually, the MYB domain is found to be conserved in plants, containing up to four imperfect repeats of about 52 amino acids each. R2R3-MYBs are characterized by two repeats, known as R2 and R3. Similar to characterized R2R3-MYBs in other plants, *A. coerulea* homologues also showed a conserved MYB domain with few insertions or deletions (13%) in the repeat regions. However, the sequences outside the domain region were found to be highly divergent in length as well as in amino acid composition. The repeat regions of the MYB domain in the species contained nearly 105 residues, of which 53 residues form R2 repeat while the remaining 52 constitute R3 (Fig. 1A, B). Each repeat showed typical amino acids with a series of even distributions of three conserved residues i.e., Tryptophan [W] at positions 5, 25 and 45 in R2 (Fig. 1A). The R3 repeat showed a lower degree of conservation of Tryptophan at position Trp-58 than the Trp-77 and Trp-96 (Fig. 1B). It showed substitution at position Trp-58, which is exchanged with Phenylalanine [F] in a majority of AqcoeR2R3-MYB members followed by the presence of either Tryptophan [W] or Isoleucine [I] or Leucine [L] and the other two residues showed conserved Tryptophan residue at positions 77 and 96 (Fig. 1B). The linker region, connecting R2 and R3 repeats also showed 4 highly conserved amino acids [LRPD, Leu-49 to Asp-52] in its first half, similar to other plant MYBs (Fig. 1A). The web logos also suggest that most conserved residues lie between the second and third Tryptophan in the R2 and R3 repeat as compared to conserved residues that lie between the first and the second conserved Tryptophan in R2 and R3 repeats (Fig. 1A, B).

Apart from highly conserved hydrophobic Tryptophan (W) residues in the R2 and R3 repeat regions, we identified a few other conserved residues in the MYB domains of *A. coerulea*. In particular, these highly conserved residues lie at N- and C-terminal ends of each repeat region(Fig. 1A, B). Amongst the three helices of each repeat region, the third one has been found most conserved, indicating its importance for DNA binding activity. It has been previously demonstrated that the third helices of both R2 and R3 come in contact with each other to bind to specific base sequences in the DNA major groove (Ogata et al. 1994). Due to its important role, the third helices have remained conserved across different plant species during MYB evolution (Li et al. 2016). Furthermore, the linker region between the R2 and R3 repeats also promotes stable interaction of protein-DNA complexes. The linker region is highly sensitive to mutation such that a few substitutions can lead to loss of DNA binding ability (Dias et al. 2003; Heine et al. 2004). Amongst all the identified members, a single homologue showed a substitution at Pro51 by Glycine (G) in the linker region that may affect its DNA-binding ability.

### Phylogenetic and conserved C-terminal motif analysis

**Fig. 2:**
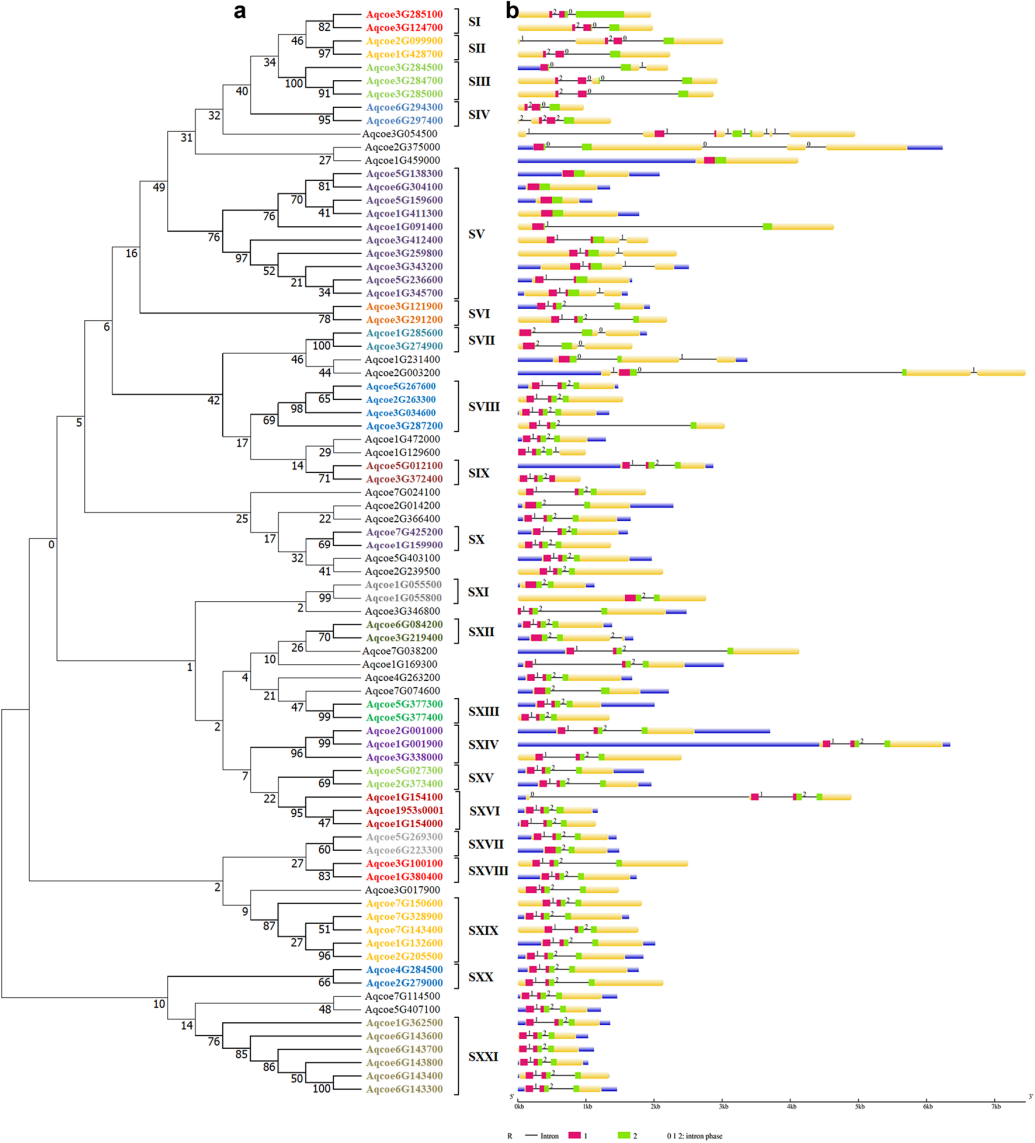
Phylogenetic relationship and gene organization of *A. coerulea* R2R3-MYB genes:** A** Phylogenetic tree of *A. coerulea* R2R3-MYB members based on full length protein sequence constructed through unrooted neighbor-joining method in MEGAX. The tree clustered into 21 subgroups named as SI to SXXI based on bootstrap value of more than 60%, **B** Exon–intron structure, intron phases and conserved MYB domain of AqcoeR2R3-MYBs. Exons are shown by yellow boxes, R2 as red boxes and R3 as green box, introns as black lines along with intron phases (0, 1, 2) and untranslated regions as blue boxes. The length of each AqcoeR2R3-MYB gene can be estimated using scale at the bottom

The NJ phylogenetic reconstruction grouped 82 AqcoeMYBs complete protein sequences into 21 subgroups with at least 70% bootstrap support. Most of the clades showed gene pairs with a high bootstrap value of 90% (2, 3, 4, 7, 11, 13, 14, 21) (Fig. 2A). The topology of the tree remained similar when the conserved domain sequence region (MYB domain) was used for phylogenetic analysis (Supplementary Fig. 1).

To infer the putative role of identified AqcoeMYBs in the study, phylogenetic NJ tree reconstruction was performed with characterized MYBs from other plants such as *Arabidopsis*, grape, rice, poplar and maize. The phylogenetic tree resolved MYB proteins into 21 subgroups within the species and C1-C100 clades across different species (Fig. 2A, Supplementary Fig. 2). Foremost, it has been found that there are quantitative differences that exist in several clades. For example, clade 43 which includes representatives from all species showed lineage-specific expansion in *A. coerulea*. In contrast, clades such as C3 and, C85 contained a single MYB homologue of *A. coerulea* and a few clades (82, 83, 84) with a complete absence of *Aquilegia* homologues (Supplementary Fig. 2). The analysis showed a total of 15 clades with representatives from all the taxa chosen for the study which confirms that the main diversification of R2R3-MYB occurred before the monocot and dicot split (Chang et al. 2020). This also provides an excellent reference to explore the potential gene functions of AqcoeR2R3-MYBs as these proteins are clustered with *A. thaliana* functional clades. However, there were 16 AqcoeR2R3-MYB protein sequences which did not fall into any of the clades. This indicates that these species-specific genes were either lost in *A. thaliana* and other plants included in the phylogenetic analysis or acquired in the *Aquilegia* during its genome evolution. It should be noted that adding more R2R3-MYB genomic sequences from other taxa could also resolve the placement of these 16 AqcoeR2R3-MYBs in the present phylogenetic tree. Further, twenty clades were observed within which either rice or grape or *A. thaliana* or *P. trichocarpa* sequences were absent (C5, 6, 11, 19, 20, 21, 47, 68, 72, 73, 74, 77, 79, 86, 87, 89, 90, 93, 94 and 97). Meanwhile, some of the clades found were specific to each species for example, 14 clades contained only *A. thaliana* protein sequences (C17, 26, 27, 54, 55, 63, 71, 75, 82, 83, 84, 92, 96, 99), 10 clades with *O. sativa* (C2, 4, 7, 10, 13, 16, 65, 70, 76, 78, 91), 11 clades with *P. trichocarpa* (C8, 33, 35, 9, 40, 49, 50, 51, 56, 57, 61) and 3 clades with *V. vinifera* (C22, 41, 42). So, it is quite possible that R2R3-MYBs might have experienced another round of major independent expansions in each of these selected lineages during the evolution of angiosperms which might contribute to their functional divergence (Gates et al. 2016; Wu et al. 2022) Furthermore, 23 clades showed complete absence of AqcoeMYBs sequences (C12, 15, 23, 24, 25, 28, 29, 31, 32, 34, 36, 37, 38, 53, 58, 59, 60, 62, 64, 67, 69, 80, 98), The possible explanation for low number of AqcoeR2R3-MYBs could be due to accumulation of repetitive sequences in the *Aquilegia* chromosomes that might have led to genic losses (Filiault et al. 2018).

**Fig. 3:**
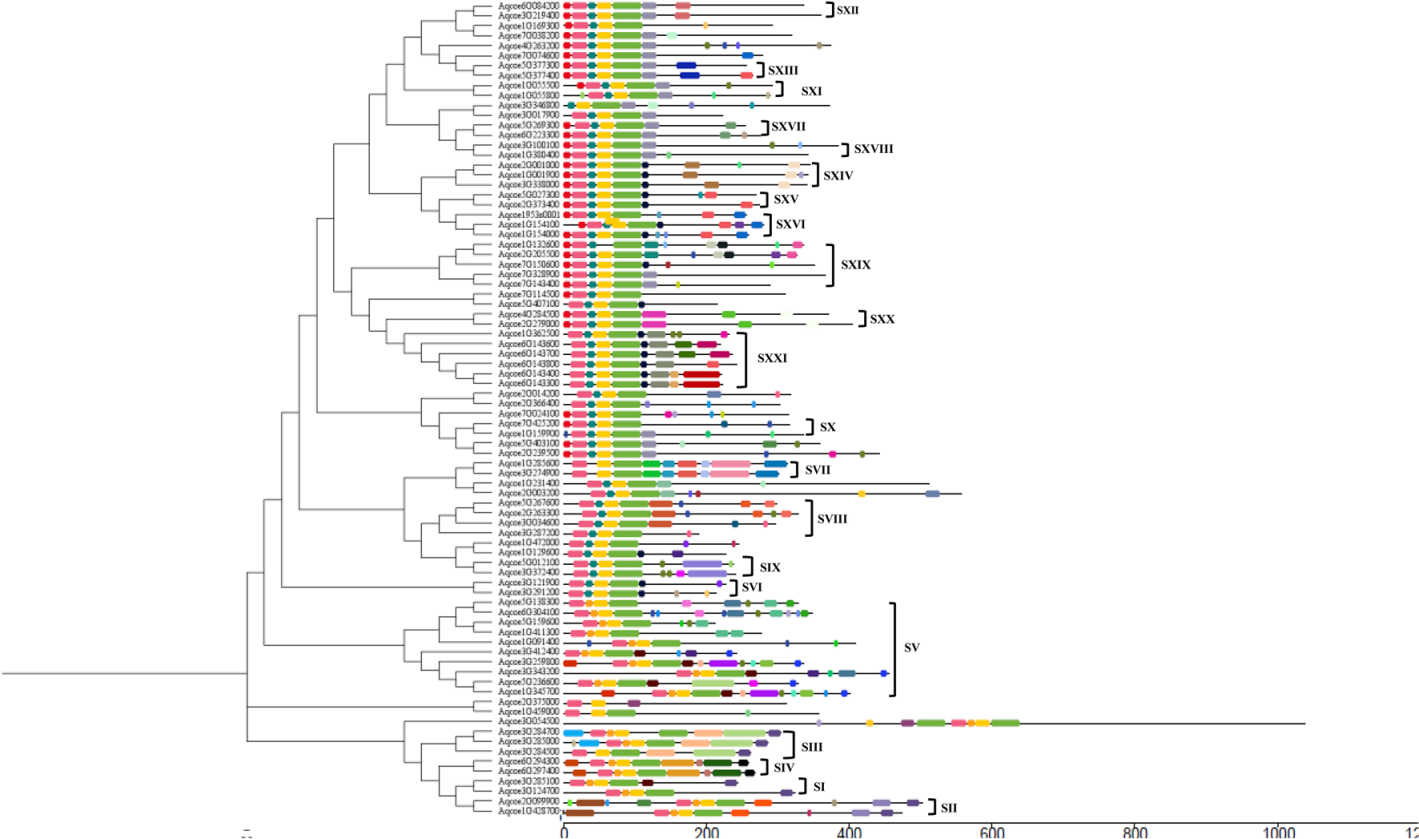
Conserved motifs analysis across *A.coerulea* R2R3-MYB genes: Different conserved protein motifs observed (right side) in 21 subgroups of *A. coerulea* phylogenetic tree. The motifs were detected using TBtools and graphically represented as colored boxes. Different color boxes represent different motifs

To further test the reliability of phylogenetic classification of AqcoeR2R3-MYBs, conserved non-MYB C-terminal motifs were predicted and analysed (Fig. 3). A minimum of 100 motifs were predicted for 82 AqcoeR2R3-MYB proteins through the online MEME suite and visualized by Tbtools (Fig. 3). The length and sequence of all predicted motifs are provided in Supplementary Table 4. The composition and distribution of motifs within subgroups were found to be relatively more conserved than between subgroups, hence supporting the phylogenetic classification. There are four motifs (1–4) seen at the N-terminal end shared by almost all members of AqcoeR2R3-MYBs. Otherwise, most of the motifs present at the C-terminal region were exclusive only to subgroups. For example, in subgroup IV, all the R2R3-MYB proteins contained motifs 1, 2, 3, 4, 23, 30, 42, and 70, and in subgroup 7, all the proteins contained motifs 1, 2, 3, 14, 24, 25, 27, 46 and 54 (Fig. 3). However, some motifs appear to be unique to subgroups. For example, motif 20 was only identified in subgroup XIV while motif 11 was only restricted to subgroup XXI (Fig. 3).

Besides, the analysis also facilitates the identification of functional motifs outside the MYB domain that are necessary for transcriptional activity (Stracke et al. 2001; Jiang et al. 2004; Muñoz-Gómez et al. 2021). Few subgroup-specific motifs have been previously characterized as additional functional domains that contribute to regulatory specificity across the plant kingdom (Stracke et al. 2001; Jiang et al. 2004; Muñoz-Gómez et al. 2021). One such example of a conserved sequence motif is (AQWESARxxAExRLxRES) in MIXTA-like MYB proteins found across different taxa (Stracke et al. 2001; Jaffé et al. 2007). Another example of a conserved motif (KPRPRS/TF) which has been regarded as predictive of SG6 homologs in most eudicot sequences (Muñoz-Gómez et al. 2021). Therefore, the MEME online prediction tool was used to identify conserved motifs related to function in AqcoeR2R3-MYB members. The present study revealed the presence of a few important functional motifs that can serve as important indicators for predicting the biological functions of AqcoeR2R3MYBs. For example, six R2R3MYB sequences from subgroup XXI (Aqcoe1G62500, Aqcoe6G143600, Aqcoe6G143700, Aqcoe6G143800, Aqcoe6G143400 and Aqcoe6G143300) also shows the presence of same motif composition (KPQPLTF) which strongly supports a similar role of these members in anthocyanin biosynthetic pathway in *A. coerulea* (Supplementary Fig. 3).

### Gene structure and physicochemical analyses

The exon–intron boundaries, numbers of introns and presence of different intron phases in a gene provide important information on the evolution of gene families (Long et al. 1995). In the present study, *A. coerulea* R2R3-MYBs showed gene lengths within a range of 924 bp to 9485 bp (Supplementary Table 3). However, more than 60% of members showed an average gene size of nearly 2 kb. The maximum gene size observed was 9.48 kb in one R2R3-MYB followed by 8 kb (1), 7 kb (1) and 5 kb (4). The range of CDS and amino acid lengths observed for R2R3-MYBs was 570–3420 bp and 190–1140 AA, respectively (Supplementary Table 3).

**Fig. 4:**
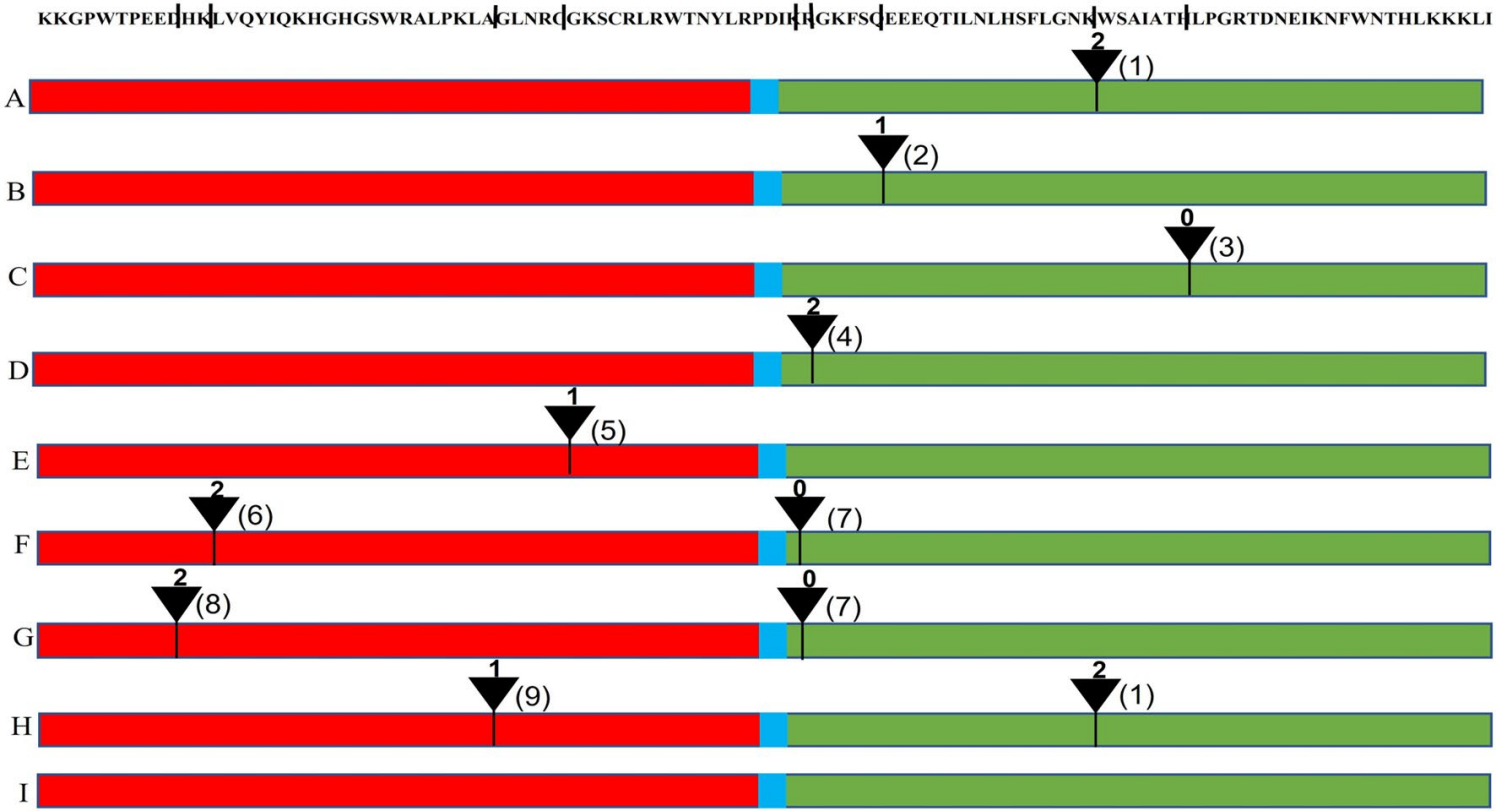
Intron-distribution patterns within R2R3-MYB domain of *A. coerulea* genes: Nine different splicing patterns across AqcoeR2R3MYBs are shown here**.** Red, blue and green rectangles represent R2, linker and R3 regions, respectively. According to the relative position and different phases of intron within the domain, the splicing patterns are designated from A-I. The black triangle depicts the location of intron, number above the triangles indicate the intron phase (0, 1, 2) and number right to the triangle shows different position (1–9)

To gain knowledge on AqcoeR2R3-MYB gene structure, exon–intron distribution and number of introns, the position and intron phases were analysed (Figs. 2B, 4). The exon number ranged from one to seven with a majority of the members showing three exons (74.3%) which appears to be a favourable state of MYB genes. Few members possessed either two (8.5%) or four exons (7.3%). AqcoeMYB33 was found to be the only member with seven exons. It is also depicted in the gene structure that in most MYBs, the domain is encoded mainly by three exons, i.e., R2 by the first and second and R3 by the second and third exons (Fig. 2B). This pattern deviates in genes with two exons and multi-exons (more than three exons) (Fig. 2B). Furthermore, the number of introns in the species ranged from as low as 0 to as high as 9. However, the majority of R2R3-MYB members (84%) showed two introns in the gene. The number of intron-less genes were five in total out of 82 genes. There are also a few genes observed with one intron (7), three introns (7) and nine introns (1). The analysis further revealed that there is no bias in the genomic distribution of intron-containing and intron-less R2R3-MYBs (Fig. 2B). These observations validate and further strengthen our classification of AqcoeMYBs and indicate that there is high structural conservation of MYB genes across the plant kingdom (Du et al 2015; Wu et al. 2022). The observed deviations from the majority case are not random but specific insertions or deletions in a lineage and retained in the genome during the course of evolution.

Alternative splicing (AS) increases the transcript diversity and possibly leads to protein evolution (Marquez et al. 2012). There is one factor, i.e., intron phase, that determines which exons are targeted for AS. Intron phase determines exon shuffling potential which in turn determines protein domain shuffling and eventually protein evolution (De souza et al. 1996). Usually, exons that are subjected to AS are always flanked by same-phase introns viz., symmetrical exons. In contrast, exons flanked by different-phase introns cannot be alternatively spliced as they cause frameshifts resulting in premature stop codons and truncation of the ORFs (Magen and Ast 2005; Roy and Penny 2007). In the present study, nine different intron splicing patterns were recognized based on the position of introns and phases within the MYB binding domain (Fig. 4A–I). The most common splicing pattern observed was with two introns distributed at two conserved positions (Fig. 4H), which accounted for 59.7% of R2R3-MYBs. The less common ones showed either a single intron present at varying positions within the R3 domain (13.4%) (Fig. 4A–D) or within the R2 domain (6.09%) (Fig. 4E) or no intron at all (6.09%) (Fig. 4I). Amongst all, there is one intron phase pattern (1:2) observed within the MYB binding DNA domain seen across most of the members. This intron phase pattern is also common among monocots and core-eudicots which indicates that this was established in their common ancestor. In addition, we observed six genome-specific intron phases [(2:NA); (0:NA); (1:NA); (2:0:0); (2:0); (2:2)] within the MYB DNA binding domain yet they all are rare and present in only a few selected MYBs. These may represent recent evolutionary events and need further validation.

All R2R3 homologues (82) were analysed for three physicochemical attributes such as the composition of amino acids, molecular weight (MW), isoelectric point (pI) and hydropathy (GRAVY score) that decide the three-dimensional conformation and nature of the protein and hence its function (Aftabuddin and Kundu 2007; Brown et al. 2010). The molecular weight of AqcoeMYBs showed wide distribution from as low as 21 KD to as high as 127 KD (Supplementary Fig. 4A, Supplementary Table 3). Majority of the MYBs (39%) were observed with molecular weight ranging from 30–40 KD followed by 29% of MYBs with less than 30 KD which reflects structure–function constraints. A few R2R3-MYBs showed a frequency of 13% and 7.3% with MW less than 50 and 60 KD, respectively. The extreme molecular weight was observed only for two AqcoeMYBs i.e., 118 and 127 KD (Supplementary Fig. 4A, Supplementary Table 3). The distribution of protein size of R2R3-MYBs in other plant species such as *Triticum aestivum* is comparable to what we observed in *A. coerulea* (Wei et al. 2020). While the drastic reduction of protein size to half is reported in *Ipomoea* spp. (Li et al. 2023). The availability of more information across the phylogenetic landscape, i.e., from early land plants to higher eudicots, can suggest a trend in the reduction of protein size of R2R3-MYBs during the course of evolution.

Isoelectric points of all R2R3-MYBs (82) range from 4.76 to 9.35 (Supplementary Fig. 4B, Supplementary Table 3). To be precise, 31% (26) of the R2R3-MYBs showed a pI range between 5 and 6 followed by 19% (16) with 6 < pI < 7 and 8 < pI < 9, 13.4% (11) with 9 < pI < 10. Only 7.3% MYBs displayed pI between 4–5 and 7–8 (Supplementary Fig. 4B, Supplementary Table 3). It has been suggested from proteome studies across the plant kingdom that an acidic pI is most common followed by an alkaline pI and finally a pI that is near neutral is the least common (Mohanta et al. 2019). Comparative analysis across all AqcoeMYB members revealed that the pI value for most of the AqcoeMYBs lie in the range of 4–7. None of the MYB proteins showed an extreme acidic range < 4. Across phylogeny, most homologs falling within the same subgroup share more or less similar pI range of values with a few notable exceptions. For example, subgroup III shows homologs with both acidic (Aqcoe3G285000) and alkaline range (Aqcoe3G284500, Aqcoe3G284700) (Fig. 2A, Supplementary Table 3). The varying physiochemical attributes of AqcoeR2R3-MYBs present among the members of the same subgroup may help in establishing their functional differentiation over the course of its evolution (Kiraga et al. 2007).

In addition, GRAVY values were also determined to analyze the hydrophobic and hydrophilic characteristics of the AqcoeR2R3-MYBs members. A GRAVY scores greater than zero indicates an integral membrane protein while a score less than zero indicates a soluble nature of the protein (Kyte and Doolittle 1982). In the study, the minimum and maximum GRAVY score recorded for AqcoeMYB proteins were -1.0 and -0.4, respectively (Supplementary Fig. 4C, Supplementary Table 3). This suggests that all the identified R2R3-MYB proteins are soluble proteins, one of the requirements for proteins to work as transcription factors (Katiyar et al. 2012).

### Structural analyses of AqcoeR2R3-MYB domain

**Fig. 5:**
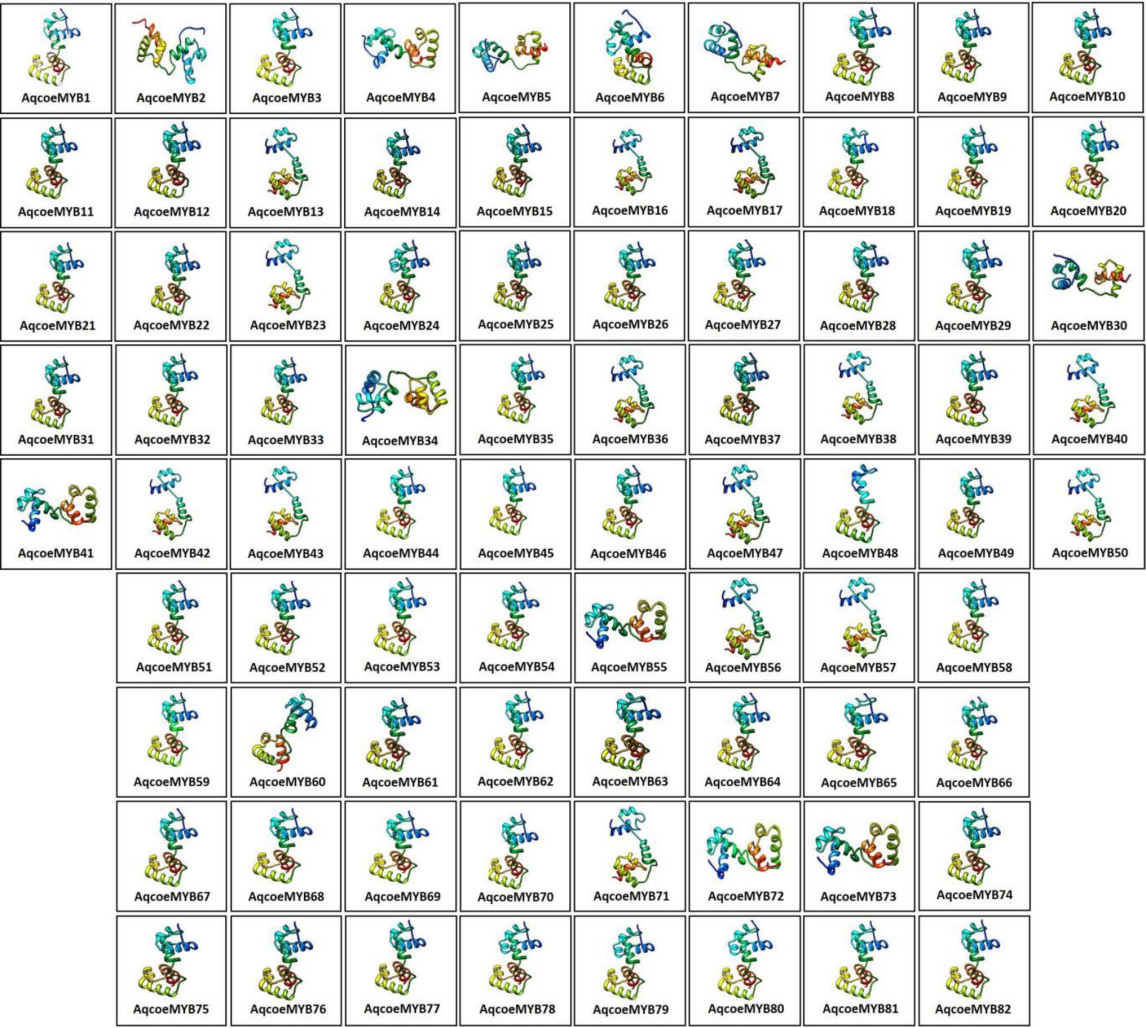
Three-dimensional structure and folding pattern of R2R3-MYB domain of *A. coerulea* proteins: Predicted protein architecture of MYB domain of all 82 R2R3-MYBs through Phyre 2 protein modelling server. Each model is shown in rainbow color from N to C terminus

All AqcoeR2R3-MYBs were analysed for their secondary and tertiary structure using the Phyre 2 server to understand the sequence-structure relationship (Fig. 5, Supplementary Table 5). The best models predicted for AqcoeMYB proteins showed query coverage ranging from 69–99% and sequence identity from 38–88% with their template sequences. Analysis of 3-D structure based on homology modelling revealed structural similarity of AqcoeMYB proteins with different pdb molecules (Nishina et al. 1989; Wang et al. 2020b). A majority of the AqcoeMYB proteins showed similarity with the c6kksA protein model/template of *Arabidopsis* R2R3 type MYB2 TF (WEREWOLF, WER). However, a few were modelled with template c1h88C, a ternary protein-DNA complex 1 and four MYBs with template c1mseC of DNA complex of the MYB DNA-binding2 domain with cooperative recognition helices (Fig. 5, Supplementary Table 5). Surprisingly, a single AqcoeR2R3MYB was modelled with template c7w59L which is a cell division cycle 5-like protein. These deviations are found random in the course of evolution, as they were not particular to any subgroup/lineage. The crystal structure of the WER complex with its target DNA has been studied by X-Ray diffraction and showed that third recognition helices of both R2 and R3 MYB repeats bind to the major groove of DNA in a sequence-specific manner (Wang et al. 2020b). The WER transcription factor has also been acknowledged in *Arabidopsis* as a MYB-related protein that regulates the expression of GLABRA2 to control epidermal cell patterning in a position-dependent manner (Lee and Schiefelbein 1999). Most of the AqcoeR2R3-MYB domains showed a moderate degree of sequence similarity with *Arabidopsis* WER indicating similarity in the mechanism of binding to its target DNA.

Overall, the MYBs contained 44–67% alpha helices in their secondary structure. Of all three helices in each repeat, the first one always had a higher number of turns followed by the third. The number of amino acids forming a helix in the R2 repeat usually ranged from 10–14, 4–9, 7–11 in 1st, 2nd and 3rd helices, respectively. Similarly, the AA in R3 repeat ranged from 13–14, 4–8, 4–16, in 1^st^, 2^nd^ and 3^rd^ helices. While the loop that connects 1 and 2 helices showed residues ranged from 3 to 8 and turns between 2 and 3 helices ranged from 5 to 11 aa in R2 repeat and corresponding aa residue numbers were 2–4 and 5–9 respectively, in R3 repeat (Supplementary Table 5). The predicted structure suggested the presence of a similar H-T-H structure in AqcoeR2R3-MYB proteins with already known *Arabidopsis* models and are therefore highly reliable. Both R2 and R3 repeats showed three alpha helices each. Other features observed from 3-D structure such as the length of each helix in both repeats, distance between R2 and R3 repeats and the position showed the more or less conserved nature of AqcoeMYBs indicating their importance in DNA binding activity. Particularly, most of the proteins showed conserved positions i.e., at the N-terminal end, as reported for other plant MYBs (Du et al. 2015). Thus, the comparative analysis of the AqcoeMYB sequence and their 3-D protein structure provides crucial information to understand structural determination.

Ancestral sequence reconstruction was also performed across the AqcoeR2R3-MYB gene family (Supplementary Fig. 5). The protein structure of the ancestral AqcoeR2R3-MYB for the entire gene family was predicted which showed similarity to template, c1mseC of DNA complex of the MYB DNA-binding2 domain with cooperative recognition helices (Supplementary Fig. 5A, Supplementary Table 5). However, the protein structure for each ancestral node of twenty-one subgroups showed similarity either to c6kksA protein model/template of *Arabidopsis* R2R3 type MYB2 TF (WEREWOLF, WER) or c1h88C, a ternary protein-DNA complex 1 or c1mseC of DNA complex of the MYB DNA-binding2 domain with cooperative recognition helices (Supplementary Fig. 5A, Supplementary Table 5). To understand the cause of structural variation in extant R2R3MYB homologues, we superimposed the representative structures of subgroup (I, II and VI) over the ancestral protein sequence in Chimera (Supplementary Fig. 5B). We observed deviations due to changes in second helices or turn between second and third helices of R2 domain or changes in loop between first and second helices of R2 domain. The secondary structure alignment showed that members of subgroup 1, 3, 4 and 5 showed strong similarity to template c1h88C, which is based on the 3R-MYB gene. The secondary structure alignment of the c1h88c template with these R2R3-MYB subgroup members revealed that the origin of these members might be due to the ancestral loss of one repeat in the 3R-MYB sequence.

### Chromosomal distribution and duplication analysis of *R2R3-MYB* genes

It is unclear what caused the expansion of the R2R3-MYB family in the species, thus reflecting the importance of studying the relationship between genetic divergence and duplications among its members. For this, the genomic coordinates for all the genes were retrieved from the database and mapped onto chromosomes. The results showed the distribution of MYBs on all seven chromosomes (Supplementary Fig. 6). Among all, chromosomes 1 and 2 harbour maximum number of MYBs i.e., 19 and 20, respectively, while chromosome 4 showed the lowest number of MYBs (Supplementary Fig. 6). Also, higher densities of MYBs were observed mostly on both the top and bottom of all chromosomes except chromosome 4. The central region of chromosomes lacked MYB genes except chromosomes 5 and 6 (Supplementary Fig. 6).

Based on the distribution of MYBs onto chromosomes along with phylogenetic relationships inferred from the study, a total of 18 gene clusters were identified in the *A. coerulea* genome showing homology of > 60% throughout the protein and > 90% within the MYB domain region. Eight gene clusters (AqcoeMYB65/AqcoeMYB66/AqcoeMYB67/AqcoeMYB68/AqcoeMYB69;AqcoeMYB2/AqcoeMYB3;AqcoeMYB60/AqcoeMYB61;AqcoeMYB71/AqcoeMYB72; and AqcoeMYB40/AqcoeMYB41/AqcoeMYB42) with > 90% similarity throughout the protein and > 93% within MYB domain region were physically located near to each other on chromosomes (Supplementary Fig. 6, Red coloured). The remaining ten clusters (AqcoeMYB52/AqcoeMYB27;AqcoeMYB35/AqcoeMYB45;AqcoeMYB12/AqcoeMYB39;AqcoeMYB73/AqcoeMYB55;AqcoeMYB1/AqcoeMYB20;AqcoeMYB6/AqcoeMYB24;AqcoeMYB81/AqcoeMYB9;AqcoeMYB17/AqcoeMYB23;AqcoeMYB36/AqcoeMYB43; AqcoeMYB14/AqcoeMYB66) also shared significant similarity throughout protein sequence (> 64%) and within domain region (> 98%) but were not found genetically linked on the corresponding chromosomes (Supplementary Fig. 6). Each of these ten duplicated gene pairs are shown in a different color (Supplementary Fig. 6). The Circos plot shows the occurrence of paralogs of the same gene indicating the role of extensive duplication in the expansion, evolution and functional diversification of this gene family (Supplementary Fig. 7).

To analyse selection pressure, the frequency distributions of synonymous substitutions (Ks) and the ratio with non-synonymous substitutions (Ka/Ks; ω) were calculated for seventeen AqcoeR2R3MYB paralogous pairs (Supplementary Table 6). Ka/Ks values for most of these pairs were less than 1 which indicates that they are under purifying selection except for one pair with positive selection i.e., AqcoeMYB40/AqcoeMYB41 which showed greater than 1 (Supplementary Table 6).

### Evoluton of SG6 gene lineage

**Fig. 6:**
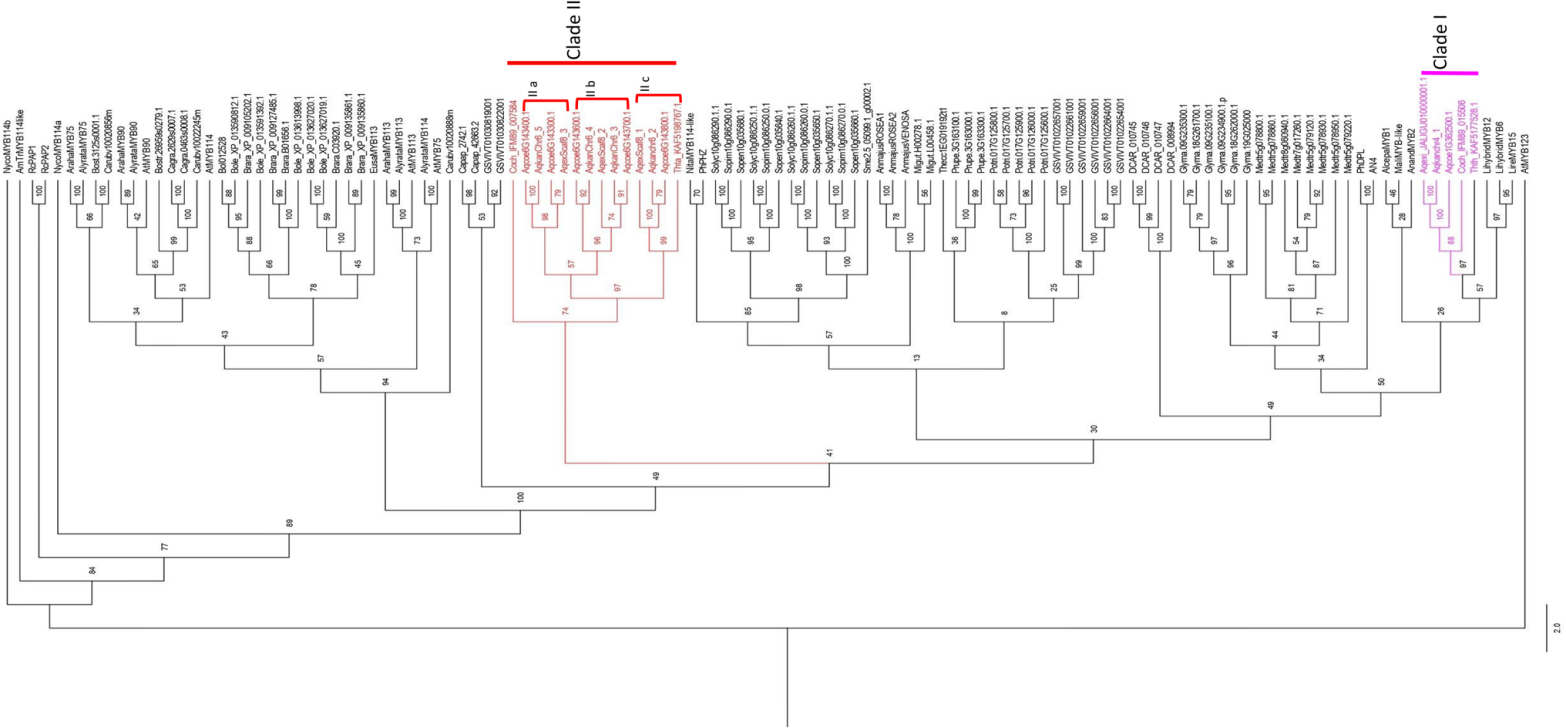
Maximum likelihood tree of the subgroup 6 R2R3-MYB genes: The selected Ranunculaceae SG6 homologues resolved into two monophyletic clades: clade I (highlighted in pink) and clade II (highlighted in red). Clade I showed a single representative of the SG6 homolog from each species used in the study (Pink). While clade II showed all species with variable number of SG6 homologs in it. Within clade II, there are three subclades (II a, II b and II c). Subclades II a and II b were only shared by three *Aquilegia* species. However, subclade II c showed representatives of each species except *C. chinensis* which falls as sister to all the three subclades

The tBLASTn searches revealed the presence of two putative homologs of SG6 gene lineage in *C. chinensis* and *T. thalictroides*. However, the number of homologues found in *Aquilegia* species were variable i.e., four in *A. eximia*, five in *A. kansuensis* and six in *A. coerulea* (Supplementary Table 2). A total of 19 sequences from Ranunculaceae family were used in ML phylogeny reconstruction along with other SG6 orthologs from basal angiosperms, monocots and eudicots (Supplementary Table 2). All 19 sequences from Ranunculaceae fall as members of SG6 lineage with AtMYB123 as outgroup (Fig. 6). All the Ranunculaceae members resolved into two monophyletic clades I and II with high bootstrap support (> 90). Clade I include a single representative of the SG6 homolog from each species used in the study (Fig. 6). However, clade II showed all species with a variable number of homologs in it. Within clade II, there are three subclades (II a, II b and II c) with bootstrap values over 95% (Fig. 6). The subclade II c also shows representatives of each species except *C. chinensis* which falls as sister to all the three subclades. Subclades II a and II b were only shared by three *Aquilegia* species. Most of the SG6 homologs of *A. coerulea* that showed local duplications in the genome were observed orthologous either to *A. eximia* or *A. kansuensis*.

The presence of diverse floral color in *Aquilegia* spp. is considered as one of the adaptive features in response to different pollinator groups (Hodges et al. 2002; Hodges and Derieg 2009). The ABP pathway involved in floral color production is found to be conserved across angiosperms (Campanella et al. 2014; Muñoz-Gómez et al. 2021). The pathway involves a series of enzymatic reactions controlled by structural genes with a few alternative branches in between for the production of non-pigmented flavonoids, including lignins, flavonols and tannins (Campanella et al. 2014; Holton and Cornish 1995). However, the ABP pathway is controlled at the transcriptional level by the regulatory component known as the MBW complex. This complex is a result of an interaction between a MYB transcription factor, a basic Helix-Loop-Helix (bHLH) factor and a WD40-repeat protein (González et al. 2008; Zhang and Schrader 2017). The R2R3-MYB genes, particularly members of the SG6 gene lineage that are known to control pigmentation in terms of color differences or spatial patterns across species (Bradshaw et al. 1995; Ding et al. 2020; Feng et al. 2018; Gates et al. 2018; Hsu et al. 2015; Lin and Rausher 2020; Lin-Wang et al. 2010; Lowry et al. 2012; Morita et al. 2006; Pérez-Díaz et al. 2016; Quattrocchio et al. 1999; Schwinn et al. 2006; Shang et al. 2011; Wu et al. 2013; Yamagishi 2016; Zufall and Rausher 2003). The present study identifies six novel SG6 genes in the *A. coerulea* genome. The phylogenetic analysis of SG6 AqcoeR2R3-MYB genes across angiosperms and their chromosomal distribution showed that the SG6 gene lineage has undergone one round of segmental duplication and three rounds of local duplications in the species. All six paralogous copies share a high level of sequence similarity with *Arabidopsis* proteins (AtMYB75, AtMYB90, AtMYB113 and AtMYB114). Furthermore, the analysis revealed that a segmental duplication event which resulted in two paralogous copies of SG6 R2R3-MYB transcription factors are also shared in other Ranunculaceae members such as *C. chinensis, T. thalictroides, A. eximia* and *A. kansuensis*, despite being phylogenetically distant from each other. This strongly suggests that segmental duplication is specific to family Ranunculaceae and all its species harbour two paralogous copies of SG6-R2R3-MYBs. The study also highlights those other four paralogous copies that arose from three local duplication events identified in the *A. coerulea* genome on chromosome 6 and shared only with two species, *A. eximia* and *A. kansuensis*. This indicates that there might be recent independent duplication events in the genus *Aquilegia* which resulted in various paralogs from other Ranunculaceae members. The present findings support the finding of Whittall et al. (2006) that the origin of diverse floral color across *Aquilegia* spp. is accomplished largely through regulatory changes rather than structural changes in the ABP pathway.

This study opens the way for further investigation of paralogous copies and their probable role in the evolution of different petal colors in the *Aquilegia*. There are also other AqcoeR2R3-MYB proteins that grouped with functional clades of *Arabidopsis* with high bootstrap value. For instance, AqcoeMYB12 and AqcoeMYB39 arose as a result of segmental duplication and share sequence similarity with male gamete cell formation protein AtMYB125 (DUO1). This suggests that both the AqcoeMYB proteins are related to male gamete cell division and differentiation (Brownfield et al. 2009). Similarly, AtMYB5 in *A. thaliana* which acts as a repressor of the anthocyanin pathway is orthologous to duplicated copies of *Aquilegia* homologues.

## Supplementary Information

Below is the link to the electronic supplementary material.

Supplementary file1 Supplementary Figure 1: An unrooted NJ Phylogenetic tree of A. coerulea R2R3-MYB members based on MYB domain region constructed through unrooted neighbor-joining method in MEGAX. Supplementary Figure 2: An unrooted NJ Phylogenetic tree reconstruction based on full length protein sequence of R2R3-MYBs from *A. coerulea* (82), *A. thaliana* (128), *Oryza sativa* (113), *Vitis vinifera* (124), *Populus trichocarpa* (200) and other eudicots (51). Supplementary Figure 3: Multiple sequence alignment of selected SG6 genes across angiosperms to show conserved motif (KPRPRS/TF) found at C-terminal end. Supplementary Figure 4:Physicochemical properties of AqcoeR2R3-MYBs analysed using computational tools A) Molecular weight, B) Isoelectric point, C) GRAVY scores. Supplementary Figure 5: A) Ancestral protein structure and folding pattern of twenty-one AqcoeR2R3-MYB subgroups were analysed using Phyre2 protein modelling server. Ancestral protein structure of R2R3 domain in A. coerulea based on 82 homologues was also predicted to understand the cause of deviations. B) Three representative protein structure of subgroups (I, II and VI) were compared with ancestral protein structure using Matchmaker chimera tool to show structural changes. Supplementary Figure 6: Physical locations mapped onto chromosome and scaffold based on the genomic coordinates obtained from Phytozome database. The paralogous AqcoeR2R3-MYB genes (local as well as segmental gene duplicates) are shown in distinct color. Supplementary Figure 7: Circos plot showing presence of ten paralogous gene pairs in *A. coerulea* connected with red lines. (PDF 4975 KB)

Supplementary file2 (XLSX 132 KB)

Supplementary file3 (PDF 543 KB)

Supplementary file4 (XLSX 43 KB)

Supplementary file5 (XLSX 50 KB)

Supplementary file6 (XLSX 20 KB)

Supplementary file7 (PDF 437 KB)

## Data Availability

All data generated during the study is included in the manuscript and its supplementary files.
