# Supplementary material for: In silico analysis of R2R3-MYB transcription factors in the basal eudicot model, Aquilegia coerulea
Source: 3 Biotech. 2024 Oct 29;14(11):284. doi: 10.1007/s13205-024-04119-y (PMC11522220; doi:10.1007/s13205-024-04119-y)
Supplement: Supplementary file 3 — Supplementary file3 (PDF 543 KB) [file 13205_2024_4119_MOESM3_ESM.pdf]

Supplementary Table 2: The list of taxa with their accession number obtained from different databases used to construct SG6 gene lineage evolution.

Taxa	Accession number	Names assigned in the tree	Database
Amborella trichopoda	AmTr_v1.0scaffold00048.187	AmtrMYB114like	Phytozome
Allium cepa	KX785130.1	AlcepaMYB1	NCBI
Anthurium andraeanum	KU726561	AnandMYB2	NCBI
Antirrhinum majus	DQ275529	AnmajusRosea1	NCBI
Antirrhinum majus	DQ275530	AnmajusRosea2	NCBI
Antirrhinum majus	DQ275531	AnmajusVenosa	NCBI
Aquilegia coerulea	Aqcoe1G362500.1	Aqcoe1G362500.1	Phytozome
Aquilegia coerulea	Aqcoe6G143300.1	Aqcoe6G143300.1	Phytozome
Aquilegia coerulea	Aqcoe6G143400.1	Aqcoe6G143400.1	Phytozome
Aquilegia coerulea	Aqcoe6G143600.1	Aqcoe6G143600.1	Phytozome
Aquilegia coerulea	Aqcoe6G143700.1	Aqcoe6G143700.1	Phytozome
Aquilegia coerulea	Aqcoe6G143800.1	Aqcoe6G143800.1	Phytozome
Aquilegia eximia	GCA_023053565.1 (33735089-33735547)	Aqexi_JALIGU010000001.1	NCBI
Aquilegia eximia	GCA_023053565.1(7175183-7176121)	AqexScaf8_1	NCBI
Aquilegia eximia	GCA_023053565.1(7187606-7188461)	AqexScaf8_2	NCBI
Aquilegia eximia	GCA_023053565.1(7206959-7207602	AqexScaf8_3	NCBI
Aquilegia kansuensis	Aqkanchr4_1(	Aqkanchr4_1	NCBI
Aquilegia kansuensis	Aqkanchr6_1(8081536-8082474)	Aqkanchr6_2	NCBI
Aquilegia kansuensis	AqkanChr6_2(8070986-8071845)	AqkanChr6_3	NCBI
Aquilegia kansuensis	AqkanChr6_3(8063350-8064086)	AqkanChr6_4	NCBI
Aquilegia kansuensis	AqkanChr6_4(8056000-8057101)	AqkanChr6_5	NCBI
Arabidopsis helleri	ArahaMYb75	ArahaMYb75	Phytozome
Arabidopsis helleri	ArahaMYB90	ArahaMYB90	Phytozome
Arabidopsis helleri	ArahaMYB113	ArahaMYB113	Phytozome
Arabidopsis lyrata	AlyrataMYB75	AlyrataMYB75	Phytozome
Arabidopsis lyrata	AlyrataMYB90	AlyrataMYB90	Phytozome
Arabidopsis lyrata	AlyrataMYB113	AlyrataMYB113	Phytozome
Arabidopsis lyrata	AlyrataMYB114	AlyrataMYB114	Phytozome
Arabidopsis thaliana	AtMYB75	AtMYB75	TAIR
Arabidopsis thaliana	AtMYB90	AtMYB90	TAIR
Arabidopsis thaliana	AtMYB113	AtMYB113	TAIR
Arabidopsis thaliana	AtMYB114	AtMYB114	TAIR
Boechera stricta	Bostr.26959s0279.1	Bostr.26959s0279.1	PlantTFDB
Boechera stricta	Bost.3125s0001.1	Bost.3125s0001.1	PlantTFDB
Brassica oleracea	Bol012528	Bol012528	Phytozome
Brassica oleracea	Bole_XP_013627020.1	Bole_XP_013627020.1	PlantTFDB
Brassica oleracea	Bole_XP_013627019.1	Bole_XP_013627019.1	PlantTFDB
Brassica oleracea	Bole_XP_013613998.1	Bole_XP_013613998.1	PlantTFDB
Brassica oleracea	Bole_XP_013591392.1	Bole_XP_013591392.1	PlantTFDB
Brassica oleracea	Bole_XP_013590812.1	Bole_XP_013590812.1	PlantTFDB
Brassica rapa	Brara_XP_009135861.1	Brara_XP_009135861.1	PlantTFDB
Brassica rapa	Brara_XP_009135860.1	Brara_XP_009135860.1	PlantTFDB
Brassica rapa	Brara_XP_009105202.1	Brara_XP_009105202.1	PlantTFDB
Brassica rapa	Brara_XP_009127485.1	Brara_XP_009127485.1	PlantTFDB
Brassica rapa	Brara.C03920.1	Brara.C03920.1	Phytozome
Brassica rapa	Brara.B01656.1	Brara.B01656.1	Phytozome
Capsella grandiflora	Cagra.2629s0007.1	Cagra.2629s0007.1	PlantTFDB
Capsella grandiflora	Cagra.0463s0008.1	Cagra.0463s0008.1	PlantTFDB
Capsella rubella	Carubv10022245m	Carubv10022245m	PlantTFDB

Capsella rubella	Carubv10020856m	Carubv10020856m	PlantTFDB
Capsella rubella	Carubv10020888m	Carubv10020888m	PlantTFDB
Carica papaya	Capap_2742.1	Capap_2742.1	Phytozome
Carica papaya	Capap_42663.2	Capap_42663.2	Phytozome
Coptis chinensis	Coch_IFM89_015506	Coch_IFM89_015506	NCBI
Coptis chinensis	Coch_IFM89_007584	Coch_IFM89_007584	NCBI
Daucus carota	DCAR_010747	DCAR_010747	Phytozome
Daucus carota	DCAR_010745	DCAR_010745	Phytozome
Daucus carota	DCAR_010746	DCAR_010746	Phytozome
Daucus carota	DCAR_008994	DCAR_008994	Phytozome
Eutrema salsugineum	EusaMYB113/114like	EusaMYB113/114like	PlantTFDB
Glycine max	Glyma.19G025000	Glyma.19G025000	PlantTFDB
Glycine max	Glyma.09G235300.1	Glyma.09G235300.1	PlantTFDB
Glycine max	Glyma.18G261700.1	Glyma.18G261700.1	PlantTFDB
Glycine max	Glyma.09G235100.1	Glyma.09G235100.1	PlantTFDB
Glycine max	Glyma.18G262000.1	Glyma.18G262000.1	PlantTFDB
Glycine max	Glyma.09G234900.1	Glyma.09G234900.1	PlantTFDB
Lilium hybrid	LihybridMYB6	LihybridMYB6	NCBI
Lilium hybrid	LihybridMYB12	LihybridMYB12	NCBI
Lilium regale	LireMYB15	LireMYB15	NCBI
Magnolia liliiflora	MaliMYB-like	MaliMYB-like	NCBI
Medicago truncatula	Medtr5g078800.1	Medtr5g078800.1	PlantTFDB
Medicago truncatula	Medtr5g078860.1	Medtr5g078860.1	PlantTFDB
Medicago truncatula	Medtr5g079220.1	Medtr5g079220.1	PlantTFDB
Medicago truncatula	Medtr5g078950.1	Medtr5g078950.1	PlantTFDB
Medicago truncatula	Medtr8g060940.1	Medtr8g060940.1	PlantTFDB
Medicago truncatula	Medtr7g017260.1	Medtr7g017260.1	PlantTFDB
Medicago truncatula	Medtr5g079120.1	Medtr5g079120.1	PlantTFDB
Medicago truncatula	Medtr5g078930.1	Medtr5g078930.1	PlantTFDB
Mimulus guttatus	Migut.H00278.1	Migut.H00278.1	Phytozome
Mimulus guttatus	Migut.L00458.1	Migut.L00458.1	Phytozome
Nicotiana tabacum	NitaMYb114-like	NitaMYb114-like	PlantTFDB
Nymphacea colorata	NycoMYB114a	NycoMYB114a	NCBI
Nymphaceae colorata	NycoMYB114b	NycoMYB114b	NCBI
Petunia hybrida	AF166702	AN2	NCBI
Petuna hybrida	HQ428106	AN4	NCBI
Petunia hybrida	HQ116169	PhDPL	NCBI
Petunia hybrida	HQ116170	PhPHZ	NCBI
Populus trichocarpa	Potri.017G125600.1	Potri.017G125600.1	PlantTFDB
Populus trichocarpa	Potri.017G125800.1	Potri.017G125800.1	PlantTFDB
Populus trichocarpa	Potri.017G125700.1	Potri.017G125700.1	PlantTFDB
Populus trichocarpa	Potri.017G125900.1	Potri.017G125900.1	PlantTFDB
Populus trichocarpa	Potri.017G126000.1	Potri.017G126000.1	PlantTFDB
Prunus persica	Prupe.3G163300.1	Prupe.3G163300.1	Phytozome
Prunus persica	Prupe.3G163100.1	Prupe.3G163100.1	Phytozome
Prunus persica	Prupe.3G163000.1	Prupe.3G163000.1	Phytozome
Ryncholaelicocattleya	MN420461	RcPAP1	NCBI
Ryncholaelicocattleya	MN420462	RcPAP2	NCBI
Solanum lycopersicum	Solyc10g086290.1.1	Solyc10g086290.1.1	Solgenomics
Solanum lycopersicum	Solyc10g086250.1.1	Solyc10g086250.1.1	Solgenomics
Solanum lycopersicum	Solyc10g086260.1.1	Solyc10g086260.1.1	Solgenomics
Solanum lycopersicum	Solyc10g086270.1.1	Solyc10g086270.1.1	Solgenomics

Solanum melongena	Sme2.5_05099.1_g00002.1	Sme2.5_05099.1_g00002.1	Solgenomics
Solanum pennellii	Sopen10g035680.1	Sopen10g035680.1	Solgenomics
Solanum pennellii	Sopen10g035640.1	Sopen10g035640.1	Solgenomics
Solanum pennellii	Sopen10g035650.1	Sopen10g035650.1	Solgenomics
Solanum pennellii	Sopen10g035660.1	Sopen10g035660.1	Solgenomics
Solanum pimplinellifolium	Sopim10g086290.0.1	Sopim10g086290.0.1	Solgenomics
Solanum pimplinellifolium	Sopim10g086250.0.1	Sopim10g086250.0.1	Solgenomics
Solanum pimplinellifolium	Sopim10g086260.0.1	Sopim10g086260.0.1	Solgenomics
Solanum pimplinellifolium	Sopim10g086270.0.1	Sopim10g086270.0.1	Solgenomics
Theobrama cacao	Thecc1EG019192t1	Thecc1EG019192t1	Phytozome
Thalictrum thalictroides	Thth_KAF5177528.1	Thth_KAF5177528.1	NCBI
Thalictrum thalictroides	Thta_KAF5198767.1	Thta_KAF5198767.1	NCBI
Vitis vinifera	GSVIVT01022657001	GSVIVT01022657001	PlantTFDB
Vitis vinifera	GSVIVT01022661001	GSVIVT01022661001	PlantTFDB
Vitis vinifera	GSVIVT01022659001	GSVIVT01022659001	PlantTFDB
Vitis vinifera	GSVIVT01022656001	GSVIVT01022656001	PlantTFDB
Vitis vinifera	GSVIVT01022654001	GSVIVT01022654001	PlantTFDB
Vitis vinifera	GSVIVT01022664001	GSVIVT01022664001	PlantTFDB
Vitis vinifera	GSVIVT01030819001	GSVIVT01030819001	PlantTFDB
Vitis vinifera	GSVIVT01030822001	GSVIVT01030822001	PlantTFDB
