# Supplementary material for: In silico analysis of R2R3-MYB transcription factors in the basal eudicot model, Aquilegia coerulea
Source: 3 Biotech. 2024 Oct 29;14(11):284. doi: 10.1007/s13205-024-04119-y (PMC11522220; doi:10.1007/s13205-024-04119-y)
Supplement: Supplementary file 7 — Supplementary file7 (PDF 437 KB) [file 13205_2024_4119_MOESM7_ESM.pdf]

Supplementary table 6: The list of gene pairs with Ka/Ks substitution values showing the model of selection.

Gene pairs	Ka	Ks	Ka/Ks
AqcoeMYB17/AqcoeMYB23	0.21395	0.35405	0.604293
AqcoeMYB36/AqcoeMYB43	0.22575	0.3786	0.577071
AqcoeMYB40/AqcoeMYB41	0.06966622	0.06175193	1.128162634
AqcoeMYB41/AqcoeMYB42	0.013728465	0.02053615	0.668502373
AqcoeMYB71/AqcoeMYB72	0.066184695	0.1592	0.415733009
AqcoeMYB60/AqcoeMYB61	0.074596635	0.1384	0.538993027
AqcoeMYB1/AqcoeMYB20	0.13345	0.27035	0.493619382
AqcoeMYB14/AqcoeMYB66	0.225197455	0.50214579	0.448470264
AqcoeMYB65/AqcoeMYB66	0.028845235	0.050354	0.572846
AqcoeMYB67/AqcoeMYB68	0.051296385	0.07541	0.680232
AqcoeMYB68/AqcoeMYB69	0.061379855	0.15345	0.399999055
AqcoeMYB55/AqcoeMYB73	0.1495	0.3327	0.449353772
AqcoeMYB35/AqcoeMYB45	0.1156	0.26735	0.432391996
AqcoeMYB12/AqcoeMYB39	0.029530115	0.057936795	0.50969535
AqcoeMYB6/AqcoeMYB24	0.09643236	0.24375	0.395619938
AqcoeMYB27/AqcoeMYB52	0.1268	0.21065	0.601946357
AqcoeMYB2/AqcoeMYB3	0.18595	0.3433	0.54165453
AqcoeMYB9/AqcoeMYB81	0.25485	0.5256	0.484874
