# Supplementary material for: In silico analysis of R2R3-MYB transcription factors in the basal eudicot model, Aquilegia coerulea
Source: 3 Biotech. 2024 Oct 29;14(11):284. doi: 10.1007/s13205-024-04119-y (PMC11522220; doi:10.1007/s13205-024-04119-y)
Supplement: Supplementary file 1 — Supplementary file1 Supplementary Figure 1: An unrooted NJ Phylogenetic tree of A. coerulea R2R3-MYB members based on MYB domain region constructed through unrooted neighbor-joining method in MEGAX. Supplementary Figure 2: An unrooted NJ Phylogenetic tree reconstruction based on full length protein sequence of R2R3-MYBs from A. coerulea (82), A. thaliana (128), Oryza sativa (113), Vitis vinifera (124), Populus trichocarpa (200) and other eudicots (51). Supplementary Figure 3: Multiple sequence alignment of selected SG6 genes across angiosperms to show conserved motif (KPRPRS/TF) found at C-terminal end. Supplementary Figure 4:Physicochemical properties of AqcoeR2R3-MYBs analysed using computational tools A) Molecular weight, B) Isoelectric point, C) GRAVY scores. Supplementary Figure 5: A) Ancestral protein structure and folding pattern of twenty-one AqcoeR2R3-MYB subgroups were analysed using Phyre2 protein modelling server. Ancestral protein structure of R2R3 domain in A. coerulea based on 82 homologues was also predicted to understand the cause of deviations. B) Three representative protein structure of subgroups (I, II and VI) were compared with ancestral protein structure using Matchmaker chimera tool to show structural changes. Supplementary Figure 6: Physical locations mapped onto chromosome and scaffold based on the genomic coordinates obtained from Phytozome database. The paralogous AqcoeR2R3-MYB genes (local as well as segmental gene duplicates) are shown in distinct color. Supplementary Figure 7: Circos plot showing presence of ten paralogous gene pairs in A. coerulea connected with red lines. (PDF 4975 KB) [file 13205_2024_4119_MOESM1_ESM.pdf]

Supplementary Fig. 1

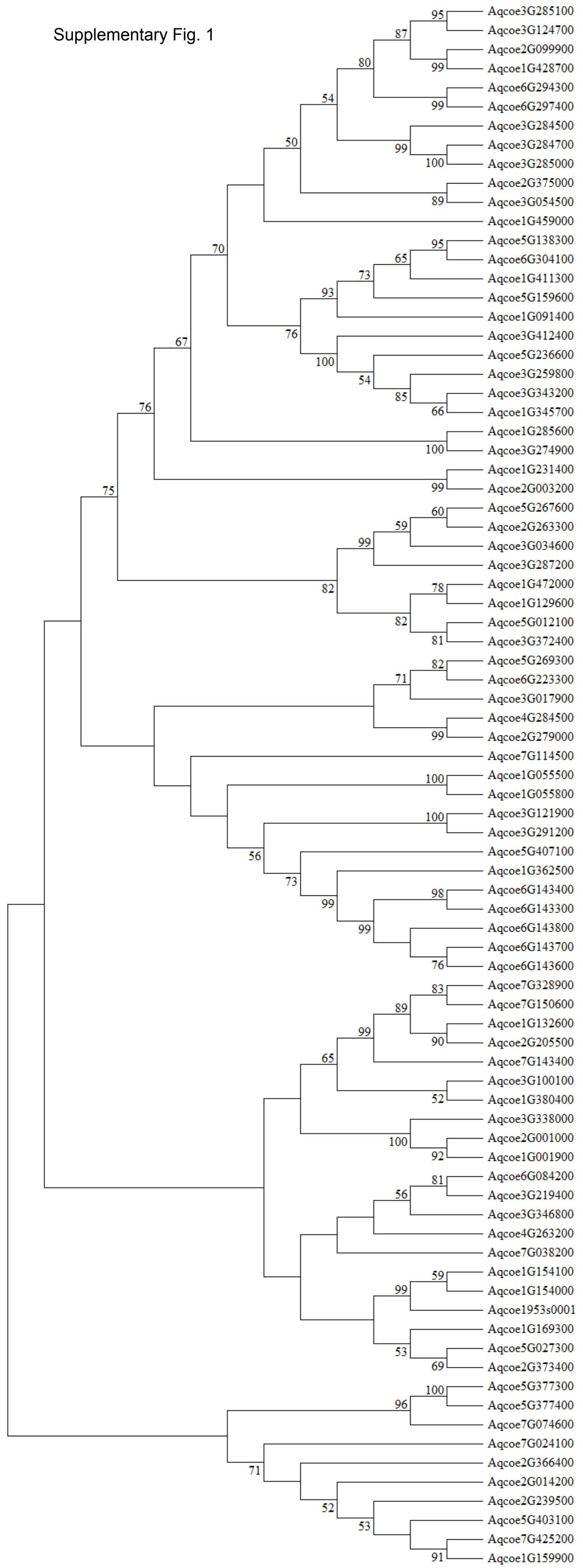

Supplementary Fig. 2

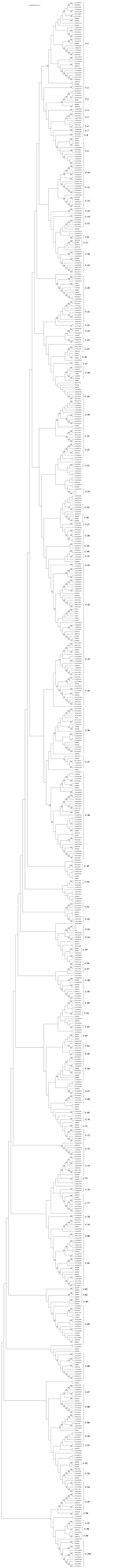

Supplementary Fig. 3

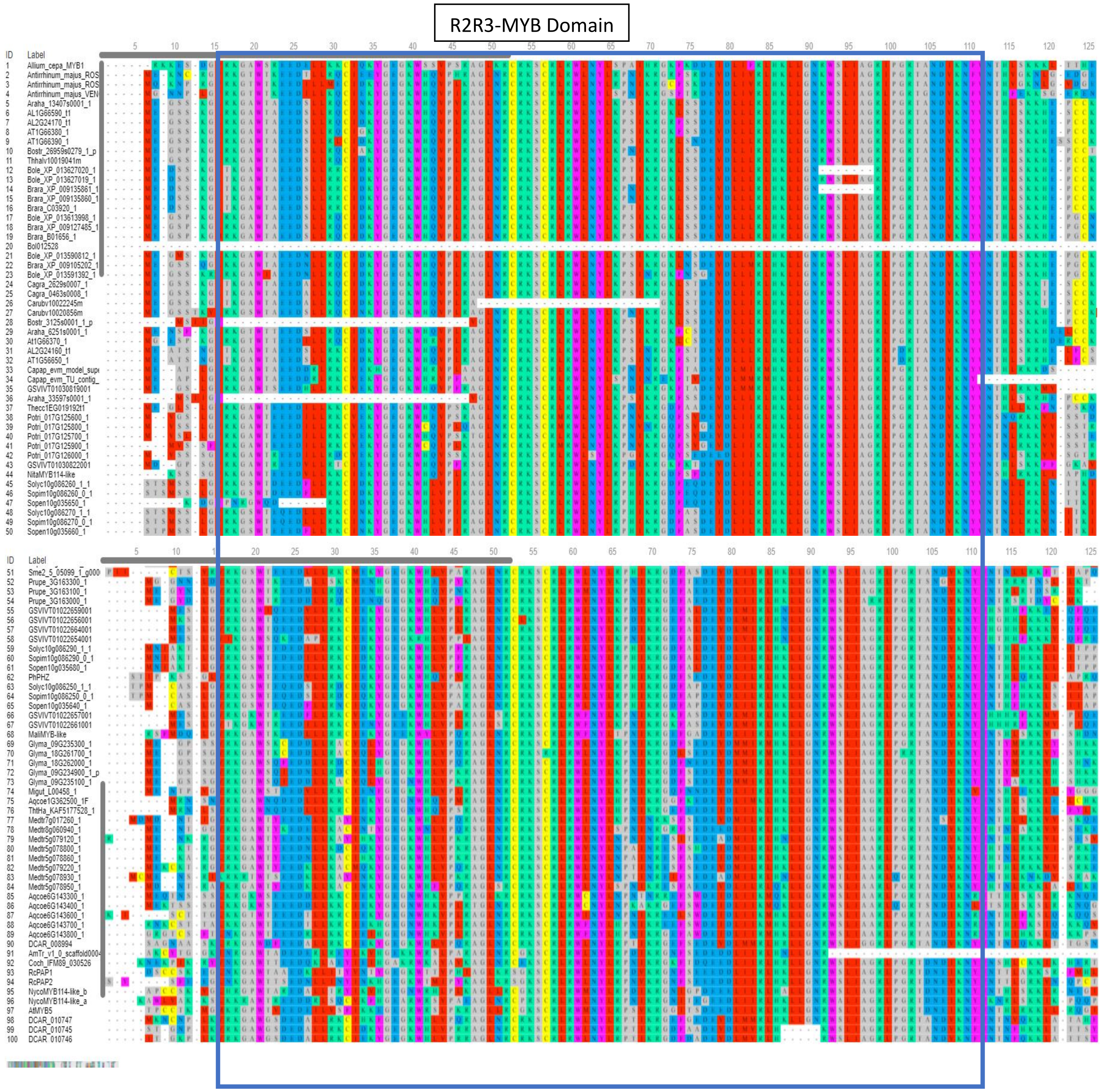

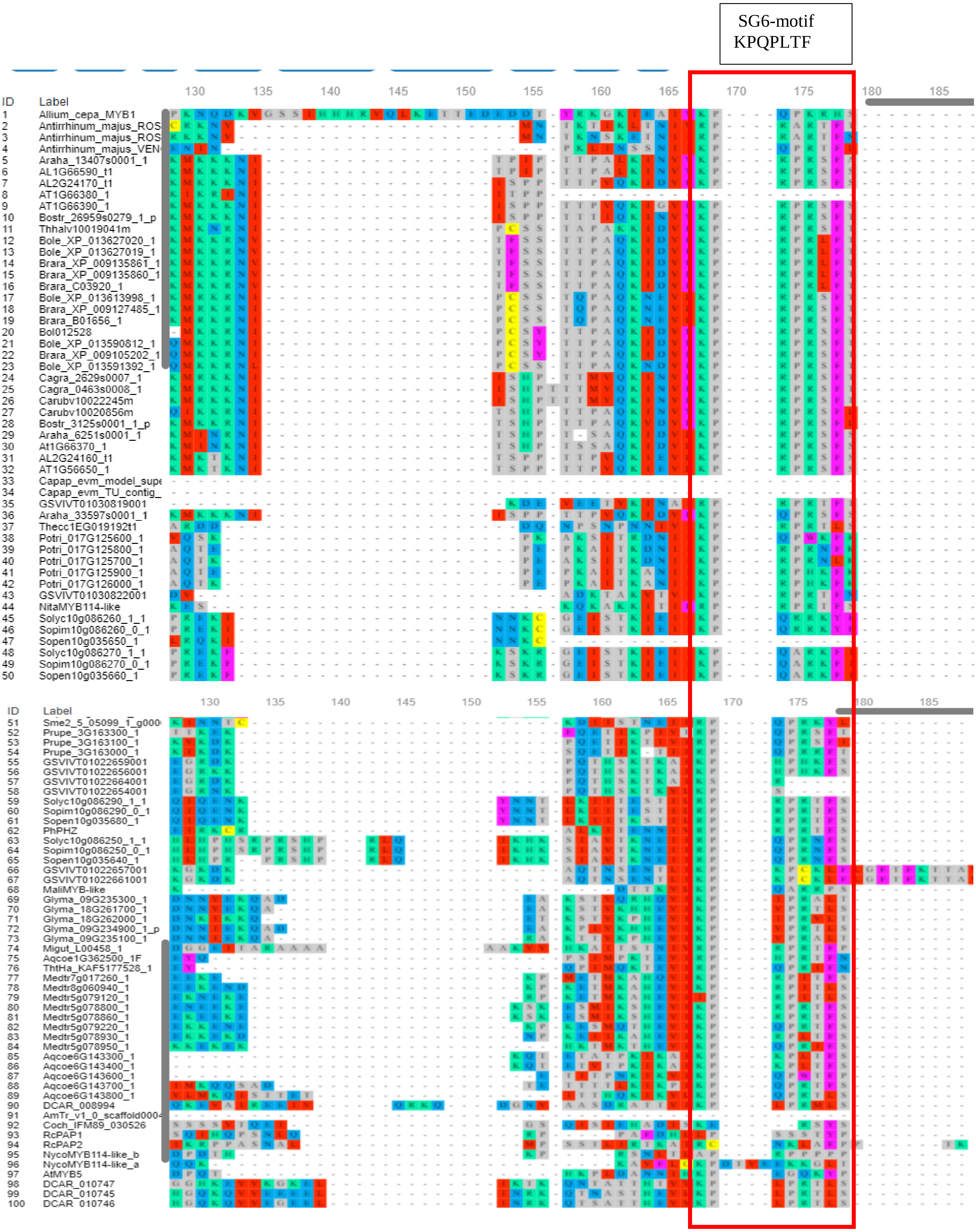
SG6-motif
KPQPLTF
ID Label
1 Allium_cepa_MYB1
2 Antirrhinum_majus_ROS
3 Antirrhinum_majus_ROS
4 Antirrhinum_majus_VEN
5 Araha_13407s0001_1
6 AL1G66590_t1
7 AL2G24170_t1
8 AT1G66380_1
9 AT1G66390_1
10 Bostr_26959s0279_1_p
11 Thhalv10019041m
12 Bole_XP_013627020_1
13 Bole_XP_013627019_1
14 Brara_XP_009135861_1
15 Brara_XP_009135860_1
16 Brara_C03920_1
17 Bole_XP_013613998_1
18 Brara_XP_009127485_1
19 Brara_B01656_1
20 Bol012528
21 Bole_XP_013590812_1
22 Brara_XP_009105202_1
23 Bole_XP_013591392_1
24 Cagra_2629s0007_1
25 Cagra_0463s0008_1
26 Carubv10022245m
27 Carubv10020856m
28 Bostr_3125s0001_1_p
29 Araha_6251s0001_1
30 At1G66370_1
31 AL2G24160_t1
32 AT1G56650_1
33 Capap_evm_model_supe
34 Capap_evm_TU_contig_
35 GSVIVT01030819001
36 Araha_33597s0001_1
37 Thecc1EG019192t1
38 Potri_017G125600_1
39 Potri_017G125800_1
40 Potri_017G125700_1
41 Potri_017G125900_1
42 Potri_017G126000_1
43 GSVIVT01030822001
44 NitaMYB114-like
45 Solyc10g086260_1_1
46 Sopim10g086260_0_1
47 Sopen10g035650_1
48 Solyc10g086270_1_1
49 Sopim10g086270_0_1
50 Sopen10g035660_1
ID Label
51 Sme2_5_05099_1_g000
52 Prupe_3G163300_1
53 Prupe_3G163100_1
54 Prupe_3G163000_1
55 GSVIVT01022659001
56 GSVIVT01022656001
57 GSVIVT01022664001
58 GSVIVT01022654001
59 Solyc10g086290_1_1
60 Sopim10g086290_0_1
61 Sopen10g035680_1
62 PhPHZ
63 Solyc10g086250_1_1
64 Sopim10g086250_0_1
65 Sopen10g035640_1
66 GSVIVT01022657001
67 GSVIVT01022661001
68 MaliMYB-like
69 Glyma_09G235300_1
70 Glyma_18G261700_1
71 Glyma_18G262000_1
72 Glyma_09G234900_1_p
73 Glyma_09G235100_1
74 Migut_L00458_1
75 Aqcoe1G362500_1F
76 ThtHa_KAF5177528_1
77 Medtr7g017260_1
78 Medtr8g060940_1
79 Medtr5g079120_1
80 Medtr5g078800_1
81 Medtr5g078860_1
82 Medtr5g079220_1
83 Medtr5g078930_1
84 Medtr5g078950_1
85 Aqcoe6G143300_1
86 Aqcoe6G143400_1
87 Aqcoe6G143600_1
88 Aqcoe6G143700_1
89 Aqcoe6G143800_1
90 DCAR_008994
91 AmTr_v1_0_scaffold0004
92 Coch_IFM89_030526
93 RcPAP1
94 RcPAP2
95 NycoMYB114-like_b
96 NycoMYB114-like_a
97 AtMYB5
98 DCAR_010747
99 DCAR_010745
100 DCAR_010746

Supplementary Fig. 4

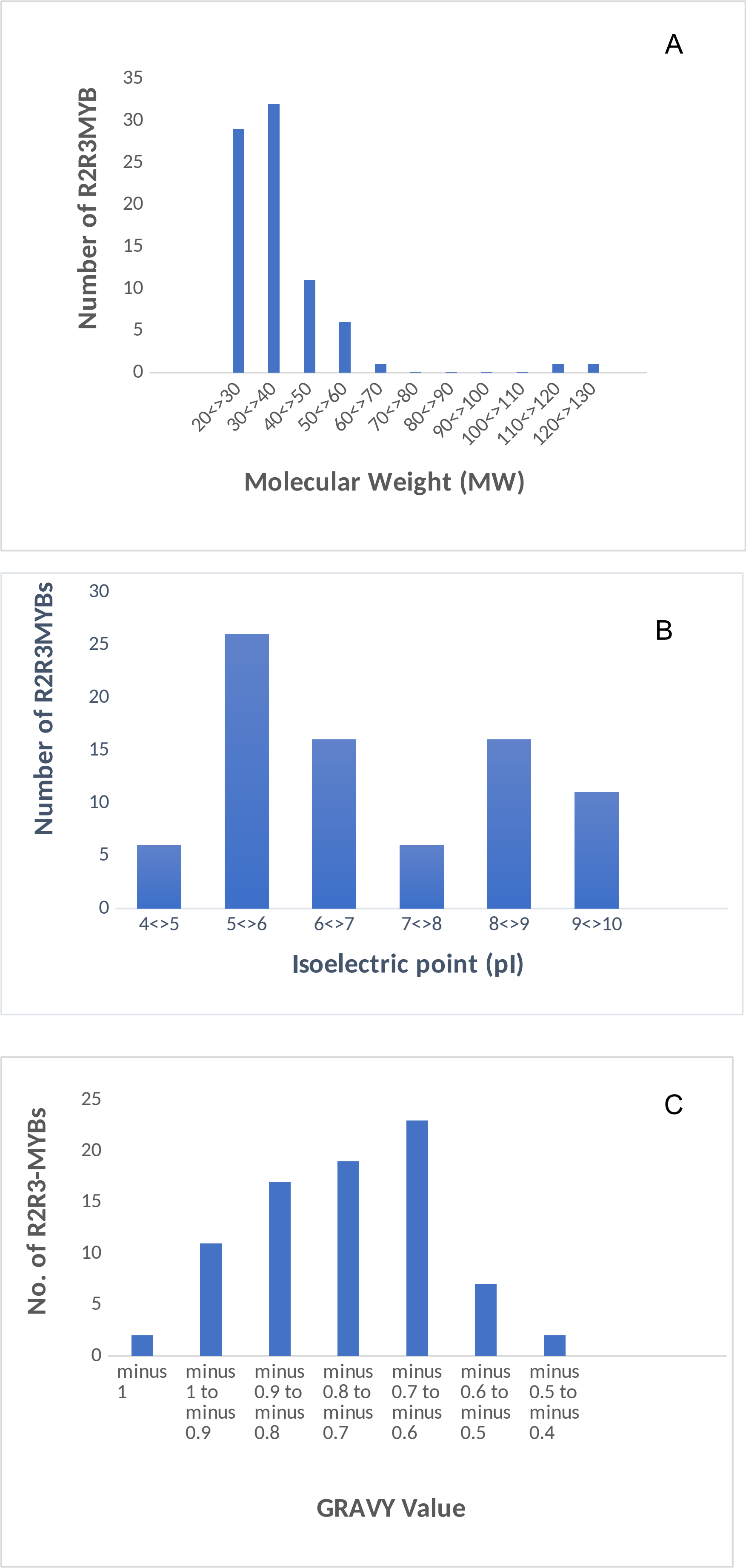

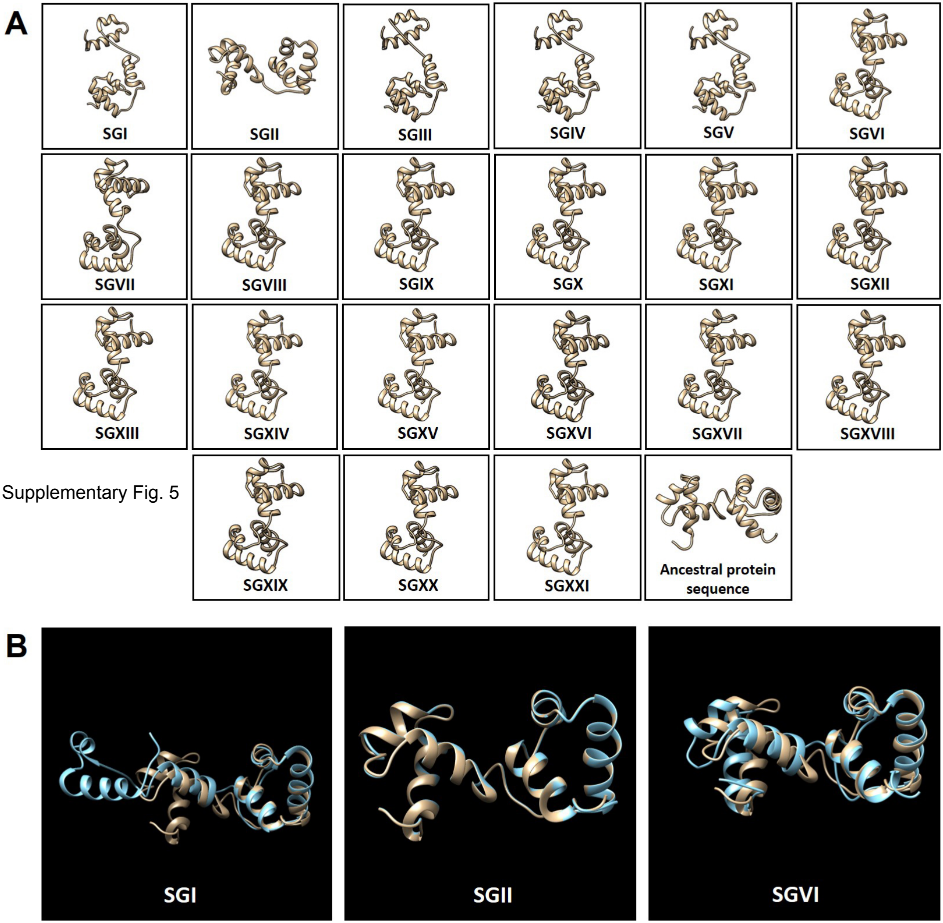

Supplementary Fig. 5

Supplementary Fig. 6

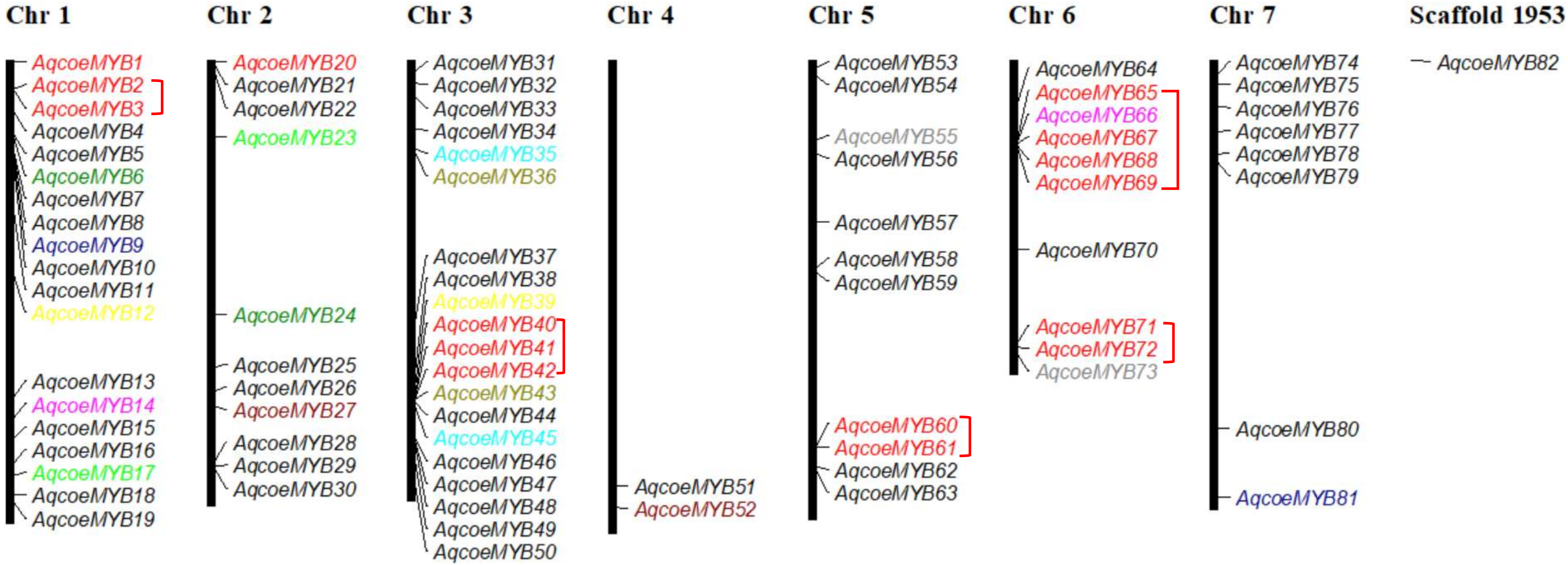

Supplementary Fig. 7

R2R3 MYB

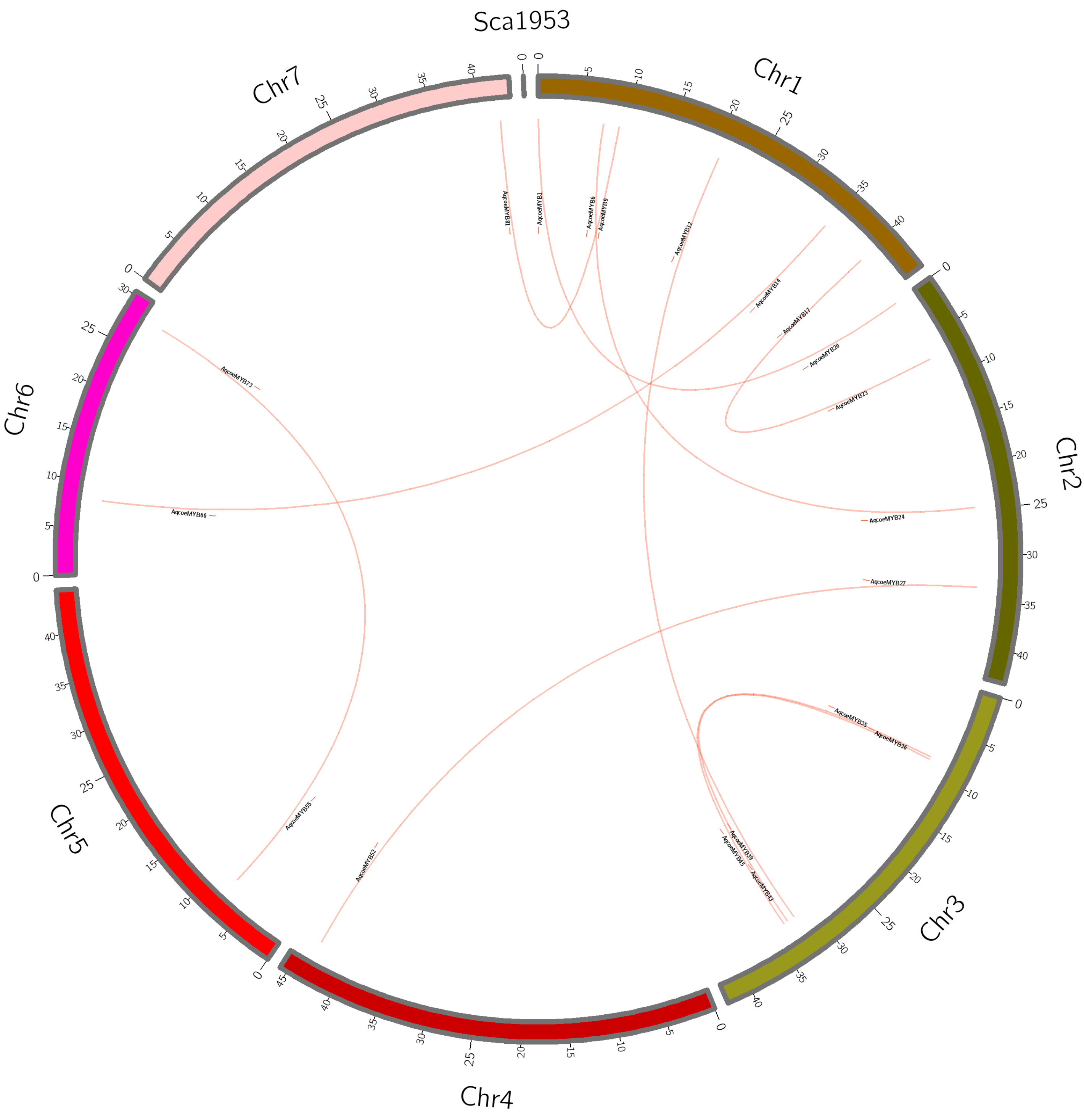

Generated by clicO using Circos tool. Powered by CodonGenomics.
